# Supplementary material for: Qualitative interview study exploring the perspectives of pregnant women on participating in controlled human infection research in the UK
Source: BMJ Open. 2023 Dec 27;13(12):e073992. doi: 10.1136/bmjopen-2023-073992 (PMC10753751; doi:10.1136/bmjopen-2023-073992)
Supplement: Supplementary data [file bmjopen-2023-073992supp001.pdf]

Neisseria lactamica in pregnancy: an interview study - Interview guide

Interview duration: 30 minutes (maximum 45 minutes)

This interview will take around 30 minutes of your time. Please stop me to ask questions if anything is not clear. This is a guide and the interviewer has flexibility to explore unexpected areas raised by the study participants. The interview guide will be reviewed after the first few interviews and small changes may be made to content and order in light of the participants' responses.

The aim of this interview is to understand what your thoughts are about a research study that we might potentially do in the future. There are no right or wrong answers. Your answers will help us to be better prepared for this study in the future.

With your permission, this interview will be audio recorded. All information you provide will be confidential, and any information that could identify you will be removed from all transcripts, and the audio will be deleted at the end of the study.

[*Note: allow prompting to clarify answers or to probe respondents thoughts further]*

Before we begin, do you have any questions or is anything not clear?

[Section 1: Understanding of meningitis]

First of all, we would like to know what you understand about meningitis. Remember there are no right or wrong answers, we are just trying to gauge what people understand.

1. Have you heard of meningitis before? Can you tell me what you understand about by the word "meningitis"?

   [Prompt: Do you worry about your child getting meningitis? Is this something you have thought about before? Do you know anyone how has had meningitis?]

Let me give you some information about meningitis to help with some further questions. Meningitis is an infection of the lining of the brain. It can be caused by bacteria that live in many people's noses and throats. Most of the time these bacteria do not cause any problems, but in some people they can, rarely, cause meningitis, and can make some people very unwell. To prevent meningitis, babies and teenagers are given vaccines. These vaccines protect against many of the bacteria that cause meningitis. However, we do not yet have vaccines that protect against all of the bacteria that cause meningitis. Furthermore, these vaccines are only given from 2 months of age, so they do not protect babies from birth.

We are hoping to carry out a research study in the future to see if we can better protect young babies from meningitis.

[Section 2: Understanding of *Neisseria lactamica* and the microbiome]

Neisseria lactamica is a type of 'good bacteria' that lives in the nose and throat. You might have heard about 'good or friendly bacteria' that can be found in some yoghurts. In healthy people with a normal immune system, 'good' bacteria do not cause any problems. Most people will carry Neisseria lactamica in their noses at some point in their lives, either as young children or as parents of young children.

We are thinking about doing a study where we would give Neisseria lactamica into the noses of pregnant women.

2. What is your first reaction to hearing this? How does this information make you feel?
3. How likely would you be to take part in a study which gave *Neisseria lactamica* into your nose whilst you were pregnant? How likely would you be to take part, with 1 being 'never' to 10 being 'definitely'? Can you explain why?

As I explained before, Neisseria lactamica is a type of 'good' bacteria that lives in the nose and throat. Having this bacteria in the nose and throat is common in childhood, with around 30% of children carrying it at any one time.

There is evidence that during outbreaks of meningitis, people who already have Neisseria lactamica in their noses are less likely to get meningitis. We have done studies in healthy adults showing that people who are given Neisseria lactamica into their noses were less likely to carry the bacteria that can cause meningitis. This is why we want to investigate Neisseria lactamica, to see whether we can protect people from meningitis, in particular young infants. We would aim to give pregnant women Neisseria lactamica into their noses at the end of pregnancy and see whether Neisseria lactamica is transferred to their babies noses in the few weeks after birth. We want to see if this changes the types of bacteria that babies carry in their noses, from less healthy types to more healthy types.

The human body is made up of more bacterial cells than human cells, the vast majority of which will never cause any problem. If someone has a severely weakened immune system, then any of these bacteria could theoretically cause infection, including Neisseria lactamica. However, there isn't any evidence that Neisseria lactamica causes infection in healthy adults.

[Section 3: Acceptability the *Neisseria lactamica* study]

4. After hearing more information, how likely is it that you would you take part in this study now? Can you rate how likely you would be to take part, with 1 being 'never' and 10 being definitely. Can you explain why?
   [Prompt: what concerns do you have? For example: Safety? If 10 people had already been given the bug would you accept it? What about if 1000 people or 10,000 people had?]

In the medical literature, there are three cases described which show Neisseria lactamica causing an infection in healthy children. These cases were reported between 1976 and 1978, and there have been no reported cases internationally since then.

5. Does this information change your mind about taking part in a research study involving *Neisseria lactamica*? Does this make the study seem more risky? What would reassure you?
6. What do you deem to be the most important risks?
   [Prompt: risk to you, risk to your baby?]
7. What other information would you require before considering participating? Which parts of the information you have heard, are most important to you, and why?
   [Prompt: Have we provided all the information that you would like to know?]
8. Having spoken about your concerns in the question before, if we provided all of this information, how likely are you now to participate in the study: 1 (never) -10 (definitely)?

Neisseria lactamica in pregnancy: an interview study. Interview Guide V1.1, 07/03/2019.
IRAS 256198 ERGO 46989

[Prompt: Which information has helped you make up your mind?]

9. What information would most encourage you to participate in this study?
10. What information would put you off participating in this study?
11. <u>We may not give *Neisseria lactamica* into the noses participants who are found already have it in their nose. If you were found to have low levels of *Neisseria lactamica* in your nose how would feel about:</u>
    a. <u>Being given the *Neisseria lactamica* spray into your nose to increase the amount that is present there?</u>
    b. <u>Not being given anything, but being followed-up as described above?</u>

<u>Study design of *Neisseria lactamica* study</u>

12. How regularly would you deem it acceptable to come to clinic for this study during pregnancy (1-2 times, 3-4 times, 5-6 times, monthly)?
    [Prompt: would you be willing to be seen more regularly if we came to your home? How else could we facilitate participation in follow-up?]
13. How often would you a be prepared to attend follow-up with your baby?
    [Prompt: once at birth, two/three/ four/six times in the first year of life. How could we facilitate participation in follow-up?]
14. In addition to any sample collected as part of routine care, what samples would be acceptable to collect from:
    a. You - blood, throat swabs, nose wash, breast milk
    b. Infant - blood, nose swabs, throat swabs

<u>Conclusion</u>

15. Do you think this study is ethical?
    [Prompt: Do you think this study is acceptable to you? if yes, why? If no, what would need to change, and what other information would you need?]
16. Do you think this study should be done?
    Prompt: if yes, why? If no, why?
17. Is there anything else you would like to share or ask?

Finally, some questions about you:

18. How many weeks pregnant are you?
19. Have you had any children before? *(if no, move to question 20)*
20. How old are your children?
21. Have you participated in research before? During pregnancy?
22. Which ethnic group do you feel you belong to? [Prompt: use codes]

Thank you

Neisseria lactamica in pregnancy: an interview study. Interview Guide V1.1, 07/03/2019.
IRAS 256198 ERGO 46989
