# Supplementary material for: Qualitative interview study exploring the perspectives of pregnant women on participating in controlled human infection research in the UK
Source: BMJ Open. 2023 Dec 27;13(12):e073992. doi: 10.1136/bmjopen-2023-073992 (PMC10753751; doi:10.1136/bmjopen-2023-073992)
Supplement: Supplementary data [file bmjopen-2023-073992supp002.pdf]

Supplementary material 1. COREQ Checklist. Consolidated criteria for reporting qualitative studies (COREQ, (1)) checklist for the study "Qualitative interview study exploring the perspectives of pregnant women on participating in controlled human infection research in the UK".

Domain 1: Research team and reflexivity

Personal Characteristics

1. Interviewer/facilitator – Which author/s conducted the interview or focus group?

   RD

2. Credentials – What were the researcher's credentials? E.g. PhD, MD

   BMBS, MSc, BMedSci(Hons), MRCPCH

3. Occupation –What was their occupation at the time of the study?

   Paediatrician on an NIHR Academic Clinical Fellowship at University Hospital Southampton and University of Southampton

4. Gender – Was the researcher male or female?

   Male

5. Experience and training – What experience or training did the researcher have?

   RD has completed research degrees (MSc and BMedSci) and NIHR Good Clinical Practice. As well as experience in controlled human infection research.

   Co-authors have experience in both clinical practice, controlled human infection research, and qualitative research.

Relationship with participants

6. Relationship established –Was a relationship established prior to study commencement?

   No

7. Participant knowledge of the interviewer – What did the participants know about the researcher? e.g. personal goals, reasons for doing the research

   Participants were made aware of the study aims and the researchers position and experience.

8. Interviewer characteristics – What characteristics were reported about the interviewer/facilitator? e.g. Bias, assumptions, reasons and interests in the research topic

   Participants were informed that the results of the interview study would inform the design of the Lactamica 9 study (2) and that results would be analysed and published.

Domain 2: study design

Theoretical framework

9. Methodological orientation and Theory – What methodological orientation was stated to underpin the study? e.g. grounded theory, discourse analysis, ethnography, phenomenology, content analysis

Thematic analysis (3)

Participant selection

10. Sampling – How were participants selected? e.g. purposive, convenience, consecutive, snowball

Purposive sampling

11. Method of approach – How were participants approached? e.g. face-to-face, telephone, mail, email

Participants were approached in person an antenatal clinic in a maternity hospital and the interview took place face-to-face at that time.

12. Sample size – How many participants were in the study?

Twelve participants

13. Non-participation – How many people refused to participate or dropped out? Reasons?

There was not dropout during the study.

Setting

14. Setting of data collection – Where was the data collected? e.g. home, clinic, workplace

Clinic

15. Presence of non-participants – Was anyone else present besides the participants and researchers?

No

16. Description of sample – What are the important characteristics of the sample? e.g. demographic data, date

All participants were pregnant and so provided relevant insights into perceptions of risk of research in pregnancy.

Data collection

17. Interview guide – Were questions, prompts, guides provided by the authors? Was it pilot tested?

An interview guide was used (see supplementary material 1) which contained verbal information and question prompts for the interview. This was designed with input from a PPI group.

18. Repeat interviews – Were repeat interviews carried out? If yes, how many?

No

19. Audio/visual recording – Did the research use audio or visual recording to collect the data?

Audio recording of interviews was performed.

20. Field notes – Were field notes made during and/or after the interview or focus group?

No. Audio recordings of interviews were transcribed post hoc.

21. Duration – What was the duration of the interviews or focus group?

Interviews lasted 30 to 60 minutes.

22. Data saturation – Was data saturation discussed?

Participants were recruited until data saturation was reached as determined by RD, TV, and CJ.

23. Transcripts returned – Were transcripts returned to participants for comment and/or correction?

No

Domain 3: analysis and findings

Data analysis

24. Number of data coders – How many data coders coded the data?

Data was coded by RD and cross-checked by, and discussed with, between RD, TV, CJ.

25. Description of the coding tree – Did authors provide a description of the coding tree?

No

26. Derivation of themes – Were themes identified in advance or derived from the data?

Themes were derived from the data and are summaries in Table 2.

27. Software – What software, if applicable, was used to manage the data?

Data was manged using NVivo (version 1.6.1), see Methods.

28. Participant checking – Did participants provide feedback on the findings?

No

Reporting

29. Quotations presented - Were participant quotations presented to illustrate the themes / findings? Was each quotation identified? e.g. participant number

Yes, quotations are provided in the Results. Participant number and relevant demographic data provided.

30. Data and findings consistent – Was there consistency between the data presented and the findings?

Quotations are presented in sections of relevant themes within the Results. These are then explored in the context of existing literature within the Discussion.

31. Clarity of major themes – Were major themes clearly presented in the findings?

Themes are summaries in Table 2 at the beginning of the Results section.

32. Clarity of minor themes – Is there a description of diverse cases or discussion of minor themes?

Subthemes are also summarised in Table 2. These are then expanded upon in the relevant sections of the discussion with pertinent quotations provided.

References

1) Tong A, Sainsbury P, Craig J. Consolidated criteria for reporting qualitative research (COREQ): a 32-item checklist for interviews and focus groups. International Journal for Quality in Health Care. 2007;19(6):349–357.
2) Theodosiou AA, Laver JR, Dale AP, Cleary DW, Jones CE, Read RC. Controlled human infection with Neisseria lactamica in late pregnancy to measure horizontal transmission and microbiome changes in mother-neonate pairs: a single-arm interventional pilot study protocol. BMJ Open. 2022;12(5):e056081.
3) Braun V, Clarke V. Using thematic analysis in psychology. Qualitative Research in Psychology. 2006;3(2):77–101.
