# Supplementary material for: Qualitative interview study exploring the perspectives of pregnant women on participating in controlled human infection research in the UK
Source: BMJ Open. 2023 Dec 27;13(12):e073992. doi: 10.1136/bmjopen-2023-073992 (PMC10753751; doi:10.1136/bmjopen-2023-073992)
Supplement: Author's manuscript [file bmjopen-2023-073992.draft_revisions.pdf]

BMJ Open

BMJ Open is committed to open peer review. As part of this commitment we make the peer review history of every article we publish publicly available.

When an article is published we post the peer reviewers' comments and the authors' responses online. We also post the versions of the paper that were used during peer review. These are the versions that the peer review comments apply to.

The versions of the paper that follow are the versions that were submitted during the peer review process. They are not the versions of record or the final published versions. They should not be cited or distributed as the published version of this manuscript.

BMJ Open is an open access journal and the full, final, typeset and author-corrected version of record of the manuscript is available on our site with no access controls, subscription charges or pay-per-view fees (http://bmjopen.bmj.com).

If you have any questions on BMJ Open's open peer review process please email info.bmjopen@bmj.com

Perspectives of pregnant women on participating in interventional controlled human infection research

Journal:	*BMJ Open*
Manuscript ID	bmjopen-2023-073992
Article Type:	Original research
Date Submitted by the Author:	23-Mar-2023
Complete List of Authors:	Dorey, Robert; University of Southampton Faculty of Medicine, ; University Hospital Southampton NHS Foundation Trust, NIHR Southampton Clinical Research Facility and NIHR Southampton Biomedical Research Centre<br>Theodosiou, Anastasia; University of Southampton, Clinical and Experimental Sciences; University Hospital Southampton NHS Foundation Trust, NIHR Southampton Clinical Research Facility and NIHR Southampton Biomedical Research Centre<br>Read, Robert; University of Southampton Faculty of Medicine; University Hospital Southampton NHS Foundation Trust, NIHR Southampton Clinical Research Facility and NIHR Southampton Biomedical Research Centre<br>Vandrevala, T; Kingston University and St George's University of London, Centre for Applied Health and Social Care Research<br>Jones, Christine; University of Southampton Faculty of Medicine; University Hospital Southampton NHS Foundation Trust, NIHR Southampton Clinical Research Facility and NIHR Southampton Biomedical Research Centre
Keywords:	QUALITATIVE RESEARCH, Maternal medicine < OBSTETRICS, Paediatric infectious disease & immunisation < PAEDIATRICS, NEONATOLOGY, OBSTETRICS, MICROBIOLOGY

SCHOLARONE™
Manuscripts

I, the Submitting Author has the right to grant and does grant on behalf of all authors of the Work (as defined in the below author licence), an exclusive licence and/or a non-exclusive licence for contributions from authors who are: i) UK Crown employees; ii) where BMJ has agreed a CC-BY licence shall apply, and/or iii) in accordance with the terms applicable for US Federal Government officers or employees acting as part of their official duties; on a worldwide, perpetual, irrevocable, royalty-free basis to BMJ Publishing Group Ltd ("BMJ") its licensees and where the relevant Journal is co-owned by BMJ to the co-owners of the Journal, to publish the Work in this journal and any other BMJ products and to exploit all rights, as set out in our licence.

The Submitting Author accepts and understands that any supply made under these terms is made by BMJ to the Submitting Author unless you are acting as an employee on behalf of your employer or a postgraduate student of an affiliated institution which is paying any applicable article publishing charge ("APC") for Open Access articles. Where the Submitting Author wishes to make the Work available on an Open Access basis (and intends to pay the relevant APC), the terms of reuse of such Open Access shall be governed by a Creative Commons licence – details of these licences and which Creative Commons licence will apply to this Work are set out in our licence referred to above.

Other than as permitted in any relevant BMJ Author's Self Archiving Policies, I confirm this Work has not been accepted for publication elsewhere, is not being considered for publication elsewhere and does not duplicate material already published. I confirm all authors consent to publication of this Work and authorise the granting of this licence.

For peer review only

Title

Perspectives of pregnant women on participating in interventional controlled human infection research

Robert B Dorey, Anastasia A Theodosiou, Robert C Read, Tushna Vandrevala, Christine E Jones

Affiliations:

Robert B Dorey BMBS, MSc, BMedSci, MRCPCH: Faculty of Medicine, University of Southampton; and NIHR Southampton Clinical Research Facility and NIHR Southampton Biomedical Research Centre, University Hospital Southampton NHS Foundation Trust, Southampton, United Kingdom

Anastasia A Theodosiou MA, MPhil, MBBChir, MRCP: Clinical and Experimental Sciences; and NIHR Southampton Clinical Research Facility and NIHR Southampton Biomedical Research Centre, University Hospital Southampton NHS Foundation Trust, Southampton, United Kingdom

Robert C Read MD, FRCP, MBChB, BMedSci, FIDSA, FESCMID: Faculty of Medicine and Institute for Life Sciences, University of Southampton; and NIHR Southampton Clinical Research Facility and NIHR Southampton Biomedical Research Centre, University Hospital Southampton NHS Foundation Trust, Southampton, United Kingdom

Tushna Vandrevala PhD, MSc, BA, CPsychol: Centre for Applied Health and Social Care Research, Faculty of Health, Social Care and Education, Kingston University and St George's University of London, London, United Kingdom

Christine E Jones BMedSci, BMBS, PgCertHBE, FRCPCH, FHEA, PhD: Faculty of Medicine and Institute for Life Sciences, University of Southampton; and NIHR Southampton Clinical Research Facility and NIHR Southampton Biomedical Research Centre, University Hospital Southampton NHS Foundation Trust, Southampton, United Kingdom

Corresponding author:

Robert B Dorey: R.Dorey@soton.ac.uk

For peer review only

Abstract

Introduction:

Pregnant women have been historically excluded from interventional research. While recent efforts have been made to improve their involvement, there remains a disparity in the evidence base for treatments available to pregnant women compared with the non-pregnant population. A significant barrier to the enrolment of pregnant women within research is risk perception and a poor understanding of decision-making in this population.

Objective:

Assess the risk perception and influences on decision-making in pregnant women, when considering whether to enrol in a hypothetical interventional research study.

Design:

Semi-structured interviews were undertaken, and thematic analysis was undertaken of participant responses.

Participants:

12 pregnant women were enrolled from an outpatient clinic.

Results:

Participants were unanimously positive about enrolling in the proposed hypothetical interventional study. Risk perception was influenced by potential risks to their fetus, and their previous experiences of healthcare and research. Participants found the uncertainty in quantifying risk for new research interventions challenging. They were motivated to enrol in research by altruism and found less invasive research interventions more tolerable.

For peer review only

Conclusion:

It is vital to understand how pregnant women balance the perceived risks and benefits of interventional research. This may help clinicians and scientists better communicate risk to pregnant women, and address the ongoing underrepresentation of pregnant women in interventional research.

Strengths and Limitations

- Relatively small sample size but semi-structured interview format allowed for rich data to be gleaned and provided detailed themes which were examined.
- Conducted with the group of interest, engaging individuals for whom pregnancy-related research is most important.
- Participants had gained experience throughout pregnancy in weighing decisions and balancing medical decisions, allowing for excellent engagement in discussion about study enrolment.
- Participants had relatively high educational attainment and so may not be representative of the broader population.

Key words

Pregnancy, neonate, human challenge, qualitative, thematic analysis, interview, risk perception, decision-making, research participation

Background

Interventional research in pregnant women

For peer review only

Interventional research in pregnancy has remained contentious since the formalisation of human research ethics in the mid-20th Century (1,2). Although neither the Nuremberg Code (1947) nor the World Medical Association's Declaration of Helsinki (1964) explicitly referred to pregnant women, their participation in interventional research was restricted following a number of highly publicised cases of harm caused by medical interventions in pregnancy (1). For instance, the use of thalidomide in pregnancy resulted in many thousands of congenital anomalies and infant deaths in the 1960s, while antenatal use of diethylstilbestrol was recognised as a cause of cancer in women and their female offspring in the 1970s (1–3). Due to perceptions of greater susceptibility to harm, pregnant women were increasingly excluded from interventional research, and even reclassified as a "vulnerable population" by the U.S. Department of Health and Human Services in 1975 (1,2).

The 1980s saw a shift towards greater inclusion of pregnant women in research studies. With dramatically increasing medical costs in the United States, research offered the public the possibility of subsidised medical care (1). Furthermore, before anti-retroviral therapies were made widely available for the treatment of HIV, clinical research participation was viewed as a means of accessing medication for an otherwise fatal infection. As the incidence of HIV in women increased, there was a rise in public opinion that the exclusion of women from HIV research studies was discriminatory. This context increased political pressure to expand inclusion of pregnant women in interventional research (1). Subsequently, in 1985, the U.S. Public Health Service Task Force publicly recognised a lack of data specific to women's health, and Congress established a Department of Women's Health at the National Institutes for Health through the passage of the Women's Health Equality Act (1990), thus mandating research in this area (2). This new sentiment was further enforced by international guidance from Council for International Organizations of Medical Sciences (CIOMS) in 1993 characterising the exclusion of pregnant women from research as "unjust" (4).

For peer review only

Despite this progress, concerns remain among clinicians and researchers that interventional studies may pose a risk of serious harm to the mother and fetus. There is evidence that clinicians over-estimate the risk of teratogenicity caused by medications during pregnancy (5). Further, ethics committees appear more likely to perceive interventional studies in pregnancy as high risk, due to difficulties in quantifying or excluding risk of harm to the fetus (6). Indeed, a study involving interviews with ethics committee members found that many members would not question the exclusion of pregnant women from research, and felt it was easier to exclude them from interventional research and opt for observational studies instead (6). These concerns may contribute to ongoing exclusion of pregnant women from interventional research. Indeed, of 172 medications approved by the U.S. Food and Drug Administration between 2000 and 2010, 97.7% has "undetermined" teratogenicity risk, while safety data relating to pregnancy was reported as "none" for 73.3% (7). Even within the last decade, pregnant women were excluded from trials of Ebola treatment and vaccines during the outbreak of 2013-2016 (8), and from up to 80% of relevant interventional studies during the first year of the SARS-CoV2 pandemic in 2020, often without specific pregnancy-related safety concerns regarding the investigational medicinal product (9). In the absence of interventional research, many treatment decisions in pregnancy therefore rely on extrapolating from research in non-pregnant individuals and post-marketing data, which may be associated with use of outdated or inferior treatments, and even worse clinical outcomes (3,10).

Even when pregnant women are eligible to participate in interventional research, they may be reluctant to enrol. Concerns amongst pregnant women include the harm that enrolling in interventional studies may cause their fetus, and a lack of knowledge about the investigational product (11,12). Furthermore, interview studies have highlighted a perception that enrolling in research may cause participants stress, something they wish to minimise during pregnancy (12,13). This may be a legacy of the exclusion from research that pregnant women have experienced, and continues to contribute to the perception that all research in pregnancy is potentially harmful,

For peer review only

regardless of the relative risks associated with a particular intervention (11). An improved understanding of how pregnant women balance risks and make decisions regarding research involvement may allow better engagement of pregnant women by research staff and allow individuals to overcome perceived barriers.

Controlled human infection research in pregnancy

Controlled human infection (also known as human challenge) research involves exposing participants to a defined number of live micro-organisms to investigate host response, colonisation, and infection kinetics, and even the effect of antimicrobial products or vaccines. Much like research in pregnancy, this type of research presents unique ethical and practical considerations over and above those shared by all interventional research (14,15), and to date has been carried out largely in non-pregnant participants. These considerations include balancing the risk to the participant and their contacts by exposure to a potentially harmful organism, against the direct benefits to the participant and potential future health benefits for society.

Neisseria lactamica is a type of harmless commensal bacteria that has been well-characterised in human challenge research involving healthy non-pregnant adults. *N. lactamica* is found in the upper respiratory tract of up to 40% of 1-2 year old infants, and approximately 10% in adults (16,17). In the immunocompetent host, *N. lactamica* does not cause clinical disease and in controlled human infection studies has been shown to displace *Neisseria meningitidis* from the upper respiratory tract, and induce cross-reactive immunity against *N. meningitidis* (18–20). To date *N. lactamica* has been safely administered intranasally to over 400 non-pregnant adults in clinical studies.

An interventional research study is underway investigating whether nasal inoculation with *N. lactamica* in late pregnancy results in maternal and infant *N. lactamica* colonisation, and whether this

For peer review only

alters the composition of neonatal upper respiratory commensal flora (ClinicalTrials.gov Identifier: NCT04784845) (21). To inform the design of this study, we carried out semi-structured interviews with pregnant women to gain insight into their risk perception and decision-making when considering their hypothetical participation in this interventional research study. Our aim was to improve our understanding of these processes, to facilitate better engagement with pregnant women, and ultimately improve research equity for pregnant women and neonates.

For peer review only

Methods

Women were approached in antenatal clinic waiting areas at a large teaching hospital in the UK, between March 2019 and February 2020. Women were eligible if they were aged over 18 years and receiving antenatal care at the site; and were excluded if they were not able to understand written and spoken English. Informed consent was obtained from all participants. The interview study was approved by Health Research Authority (HRA) London – City & East Research Ethics Committee (Reference: 19/LO/0356).

Face-to-face, semi-structured interviews were carried out with 12 pregnant women, lasting between 30 and 60 minutes each, and all interviews were audio recorded. The interview guide consisted of open-ended questions and prompts, and follow-up questions were asked to further explore themes raised by participants (see Supplementary material 1). The interview asked participants to reflect on their hypothetical participation in a proposed study and explored participants' understanding of invasive meningococcal disease, perception of risk, and acceptability of different study designs, procedures and interventions. In particular, participants were asked to consider live bacterial inoculation using nasal drops, and collection of upper respiratory (nose, throat and mouth) swabs, breast milk and blood from themself and their newborn baby. Following the interviews, recordings were transcribed verbatim and transcripts were anonymised. No further interviews were conducted

once data saturation (the point at which each new interview produces only previously discovered data) was reached. Patient and public involvement (PPI) activities were undertaken prior to present study. These activities informed the interview study design, location of recruitment, information presented, and themes discussed.

Data was managed and analysed using NVivo (version 1.6.1). Data analysis was performed in five stages as described by Braun and Clarke (22): i) familiarisation – researchers were familiarised with data prior to analysis so that broad content was understood; ii) code generation – transcripts were summarised using codes, where each code described a segment of data using a key analytic idea; iii) searching for themes – following code generation, researchers combined different codes under broader generalised patterns, called themes; which described commonalities between codes; iv) reviewing themes – once identified, codes were better defined using central organising concepts, and these themes were reviewed to ensure that they accurately represented the data, particularly as it related to the research theme; v) defining and naming themes. Codes and themes were cross-checked, discussed, and reviewed with two other researchers (CJ, TV) to ensure accurate representation of participant responses. Themes were analysed in relation to the research question, and data interpreted in this context.

For peer review only

Results

Twelve pregnant women were interviewed regarding their perceptions of risk and their decision-making about hypothetical participation in a controlled human infection study in pregnancy. Participants were women of childbearing age (25 to 40 years) with a range of educational attainment (see Table 1). Broadly, participants were white British (11/12), and seven participants did not have any

children. The majority of participants (11/12) would, in principle, agree to participate in the interventional study proposed.

Table 1. Demographic characteristics of participants

**Characteristic**		**Participant number (%)**
Marital status	Single	7 (58)
	Married	5 (42)
Gravida	Gravida 1	6 (50)
	Gravida 2	5 (42)
	Gravida 4	1 (8)
Parity	Parity 0	7 (58)
	Parity 1	4 (33)
	Parity 3	1 (8)
Ethnicity	White British	11 (92)
	Any other Asian background	1 (8)
Time in the UK	Since birth	11 (92)
	15 years or more	1 (8)
Disability or long-term health condition	No	9 (75)
	Yes	3 (25)
Highest qualification	GCSE/BTEC or equivalent	3 (25)
	AS / A-Levels or Scottish higher or equivalent	3 (25)
	BSc / BA or equivalent	5 (42)
	PhD or equivalent	1 (8)
Previous participation in research	No	11 (92)

For peer review only

	Yes	1 (8)

Overall, our data suggests that pregnant women have a heightened perception of risk, compared with when they were not pregnant. They balanced the risks and benefits of enrolling in an interventional study in the context of their individual experiences, and were motivated by the potential for developing new treatments. The acceptability of a research study was closely linked to the interventions that would be undertaken, and participants perceived uncertainty about risk to be equivalent to a high risk of harm (see Table 2 for themes and subthemes).

Table 2. Summary of themes and subthemes present in interviews with pregnant women.

**Theme**	**Subtheme**
Heightened perception of risk during pregnancy	Gestational age changes risk perception
	Babies cannot consent
Balancing risks and benefits by pregnant women	Prior understanding and experiences influence decision-making
	Conflation of uncertainty and risk
Factors which encourage participation in research	Altruism
	Research processes are reassuring
	Acceptability of study procedures

Theme 1: Heightened perception of risk during pregnancy

Participants were concerned about the risks associated with participating in research during pregnancy. This concern was attributed to two main factors: concern for the health of their unborn baby, and that they themselves may be at higher risk by virtue of being pregnant. Women expressed

For peer review only

a heightened perception of risk while pregnant, with even a small amount of risk to their baby being intolerable, although a degree of risk to themselves was felt to be more acceptable.

> *"My first and foremost concern would obviously be the safety to my baby. And then, secondary to that, myself"* (Participant 9, 26 weeks gestation, second pregnancy)

> *"With my baby, even a small amount of risk is not tolerable"* (Participant 1, 28 weeks gestation, first pregnancy)

Subtheme 1.1: Gestational age changes risk perception

Women's perception of risk was related to the gestational timing of the proposed research study, although this effect varied between individuals. Some participants reported that they would avoid participating in research in early gestation, as they perceived greater risk of complications and felt more uncertain about their pregnancy at this time. Conversely, other participants reported greater reservations about participating later in pregnancy, as they expressed more attachment to their fetus, greater confidence that the pregnancy was going to progress to birth, and that their fetus was more like an individual (in part because they could feel it move).

> *"I suppose at 36 weeks … If anything were to go wrong, 36 weeks is a very good threshold that they can convert to be induced I suppose. But yeah, that has made me think more about participating"* (Participant 7, 26 weeks gestation, first pregnancy)

> *"If I knew there was going to be a high risk to harming the baby, then I probably wouldn't because if you're doing it later on in pregnancy. Obviously that baby is already a part of you.*

For peer review only

You can feel it move, you can feel it kick and you get used to its routine and stuff like that." (Participant 4, 34 weeks gestation, first pregnancy)

Subtheme 1.2: Babies cannot consent

For some participants, the inability of the fetus to consent to research participation was paramount when considering hypothetical involvement in the proposed study. Some suggested that, since the research could theoretically cause long-term complications, they did not feel it was acceptable to consent on the unborn child's behalf. Furthermore, the baby's inability to consent would increase their own feelings of guilt if complications or adverse outcomes arose.

"I go into it knowing about it, my baby doesn't. So, I would be more worried about what it would do to my baby." (Participant 2, 12 weeks gestation, first pregnancy)

Theme 2: Balancing risks and benefits by pregnant women

For most participants, their views on hypothetical participation in the proposed human challenge study were based on a balance of perceived benefits of enrolment against potential risks and adverse events. Being pregnant brought with it, an even greater responsibility to carefully balance these risks and benefits.

Subtheme 2.1: Prior understanding and experiences influence decision-making

Previous experiences of invasive meningococcal disease influenced how they viewed their hypothetical involvement in such research. Women with personal experience, or who knew someone

affected by the condition being investigated, gave greater weight to the importance and potential benefits of the study and were therefore generally supportive of study participation.

> *"It wasn't on my radar, until a friend of mine has recently had a scare with meningitis. Her little girl had a rash that wouldn't disappear, so they took her up to hospital. But it seemed it wasn't meningitis. Then, they kind of, it does play on your mind a bit."* (Participant 7, 26 weeks gestation, first pregnancy)

Previous experience of research or medical care also weighed greatly on participants' decision-making. More specifically, traumatic experiences, particularly related to medical procedures, increased perceived risk. For example, a previous child having a difficult experience with venesection would make a study requiring neonatal blood tests appear more risky or unacceptable.

> *"I had to take my daughter quite young for a blood test. And I remember her, she was really distressed"* (Participant 10, 33 weeks gestation, second pregnancy)

For some participants, risk perceptions about study participation during pregnancy appeared entrenched and binary; either research would provide significant benefits warranting participation, or posed significant risks precluding participation. For these individuals, perspectives on risks and benefits of research did not change during the interview, despite information and safety data provided by the research team. Further, their stance did not appear specific to this particular study, but rather applied more broadly to research participation regardless of proposed interventions or procedures. In contrast, other individuals who were undecided about hypothetical study participation at the start of the interview appeared to favour participation in the hypothetical study, following provision of further information by the interviewer.

For peer review only

“Yeah, but if you already knew, I take it as if you already know information anyway and you have an opinion about it, you're going to go for it or against it. Either way. Your mind is already made up … I think it would just reassure the ones that maybe are going to, maybe people sitting on the fence might be swayed a bit more, but the “no”s definitely wouldn’t be, I don't think it would affect, I don't think it would change their mind.” (Participant 6, 27 weeks gestation, first pregnancy)

Subtheme 2.2: Conflation of uncertainty and risk

Participants were wary of the uncertainties involved in enrolling in an interventional study, especially one that has not previously been undertaken in pregnancy. They expressed difficulty in balancing proposed benefits with the potential for unknown complications, and considered the impact on their fetus more than themselves. They perceived uncertainty about potential harm in much the same way as they would known risks. Indeed, they struggled to rationalise participation when research staff could not unequivocally guarantee no risk associated with study participation.

“Because, still, you can’t guarantee it won't cause any problems” (Participant 8, 34 weeks gestation, second pregnancy)

Specific concerns were that the intervention itself could cause harm. While participants recognised that undertaking research is required to improve care for pregnant women and neonates, some felt that participation in interventional research posed greater risk to them personally than the prospect of insufficient research. This perception was heightened as they were told there may be no direct benefits to the mother or neonate of participating.

For peer review only

"I don't know. I really don't know. I think that sitting on the fence. You would never want to see your baby ill, but you also wouldn't want to put your baby in jeopardy if this is something that didn't work... That's just the way I think about it." (Participant 1, 28 weeks gestation, first pregnancy)

Theme 3: Factors which encourage participation in research

Despite the perceived uncertainties and risks, the participants were nearly unanimous in support of hypothetical participation in such a human challenge research study. Their support was mainly related to specific features of the proposed study, as well as a desire to further the care of pregnant women and neonates in general.

Subtheme 3.1: Altruism

A significant motivator for engaging with research was altruism. There was a perception that engaging with research as a pregnant woman could improve the care available to pregnant women and neonates, providing benefit to both themselves and others. Participants identified with other pregnant women and new parents, and felt motivated to improve research to reduce the chance of other families bearing the burden of disease and illness.

"I guess helping in medical research as well, meningitis can be fatal. And if you can stop another child and another family having to go through that, that would be something nice to do." (Participant 4, 34 weeks gestation, first pregnancy)

"I just think, why wouldn't you try and help if you could?" (Participant 6, 27 weeks gestation, first pregnancy)

For peer review only

Subtheme 3.2: Research processes are reassuring

Participants were reassured by the safety procedures in place regulating clinical trial. They felt confident that inclusion and exclusion criteria meant they would not be enrolled if it were inappropriate or unsafe. Further, they took comfort in knowing that they, and their baby, would be regularly reviewed by a clinician; and that if adverse events arose (affecting other participants or themselves), they would be investigated and managed according to study procedures.

> *"And knowing all of these checks, there are going to be hurdles you have to get through to take part, is good."* (Participant 2, 12 weeks gestation, first pregnancy)

> *"I would feel happier about it now knowing there are contingencies in place."* (Participant 2, 12 weeks gestation, first pregnancy)

Subtheme 3.3: Acceptability of study procedures

The proposed study intervention and sampling procedures influenced perceived acceptability. When presented with a range of sampling procedures, participants expressed greater willingness to participate if less invasive procedures were proposed, and even appeared to perceive reduced risk regarding the study as a whole (despite not altering the proposed study intervention). In particular, participants felt that research involving needles was less acceptable than those without needles, and they described a preference for heel prick sampling over venesection to acquire neonatal blood. Moreover, the proposed intervention (inoculation with live *N. lactamica*) was considered more natural, more acceptable and less risky, if administered via nasal drops than via intramuscular injection.

For peer review only

"That would be more acceptable to me, just because, it's (heel prick blood test) quick, it's simple, and I think it's not as, I think it's not as dramatic as a baby, for a baby, but yeah, it's easier" (Participant 6, 27 weeks gestation, first pregnancy)

"I think that's fine, it's not that invasive, to you or the baby, it's just a spray. So that part wouldn't really put me off." (Participant 3, 26 weeks gestation, second pregnancy)

"Well, I guess the natural version will always be more preferable" (Participant 6, 27 weeks gestation, first pregnancy)

For peer review only

Discussion

Understanding and communicating risk is complex, and most research to date on risk perception in pregnant women has focused on lifestyle measures rather than participation in interventional research (23–26). This interview-based study identified differences in participants' conceptualisation of risk, although most participants cited uncertainty, invasiveness, or personal experience as contributors to their perception of risk. Further, many participants perceived risk as binary, rather than graduated based on exposure and study-specific considerations.

Uncertainty affects perception of risk

The challenge of understanding and communicating risk may be compounded by pregnant women's apparent conflation of risk and uncertainty. In the context of research participation, risk refers to the probability of harm following an intervention, while uncertainty relates to unknown or unexpected

consequences (which may or may not be harmful). Pregnant women often equated uncertainty with harm, viewing it as a major barrier to hypothetical participation in interventional research. This finding has been reported in past literature, with pregnant women reporting a preference to let matters take their "natural course" (12,13). Thus, they distinguish between an adverse outcome arising through inaction (i.e. choosing not to participate in research) and through an action that subsequently causes harm (i.e. agreeing to a research intervention) (13).

This conflation of risk and uncertainty may be a reflection of modern obstetrics, where, due to advances in antenatal care, so much is known about a pregnancy that uncertainty is marginalised and so the unknown becomes perceived as a risk (27,28). This misconception is pervasive and affects not only how pregnant women perceive themselves, but also how healthcare staff and researchers see them, and may thus contribute to the ongoing exclusion of pregnant women from interventional research.

Invasiveness of intervention affects perception of risk

The nature of research interventions and procedures influence how risk is perceived by potential participants. Pregnant women expressed a preference for nasal compared with intramuscular inoculation, and for heel-prick sampling over infant venesection. Past research has reported reduced likelihood of study enrolment if venesection is required in children (29), while painful interventions in general are associated with negative participant experience while enrolled in research studies (30). The reasons for aversion to needles are likely complex; however, in our participants, procedures involving needles were perceived to be more invasive, and therefore less acceptable and more risky. Furthermore, our participants felt that the proposed intervention utilised a natural process (neonatal colonisation with maternal bacterial flora), and they found this more acceptable than other interventions they were asked to reflect on, such as injectable vaccines. The preference of society for

For peer review only

natural products is well described (31,32), and it may be that using non-needle-based interventions may allay concerns and alter risk perception of interventional research in pregnancy.

Prior health-related experience affects perception of risk

The interviews demonstrated that how pregnant women perceive risk and weigh decisions is greatly influenced by their prior experiences. The effect of previous experience on risk perception has previously been described, and is maintained even if there is no effect on absolute risk; for example, in genetic diseases affecting non-familial relations (33,34). Research participants who decline to be involved in studies have reported different levels of perceived risk to those who go on to enrol in research, and the information presented does not appear to change the likelihood of enrolment (11). It appears that prior life experience is important in risk perception and decision-making, and even before participants are approached, they may be pre-determined to either enrol or not based on these experiences.

Previous studies have demonstrated that, while clinicians typically present risk numerically (in terms of incidence or odds), pregnant women perceive risk in a more individualised way, relying more on personal experience than epidemiology (35).

Making research and treatment more equitable

There has been a shift away from protectionist policies in recent years, and inclusion of pregnant women within research has improved (2). However, there remains a wide disparity between engagement of pregnant women compared with non-pregnant participants in interventional research (8,9). Even recent calls "to protect [pregnant] groups through research, not just from research" (36)

For peer review only

retain vestiges of protectionist sentiment: the focus remains on researchers and governing bodies assessing risk on behalf of pregnant women, rather than empowering women to assess risk and decide whether to enrol.

Communicating risk

We have highlighted, as have others (35,37), that understanding risk is challenging for all involved in research. Research needs be made more accessible to pregnant women by improving the appreciation of the differences in risk conceptualisation between researchers and pregnant women, and improved understanding that balancing risk may depend on personal previous experience.

Patient and Public Involvement

A major barrier for pregnant women considering whether to enrol in research are logistical difficulties (11,38). Research protocols can be too demanding for participants, especially for those in full-time employment or with caring responsibilities. Patient-focussed research can be improved by involving patients early in study design through PPI (Patient and Public Involvement) groups. In addition to addressing logistical issues, this would highlight important areas of concerns that we have found in this study regarding study procedures and allow for considered amendments to protocols; excluding procedures that do not add sufficient value in answering the research question.

Reassured by healthcare professionals

Pregnant women were reassured by the stringent requirements placed upon researchers by governing bodies. They were also encouraged that researchers thought the study was of value. Confidence in research staff is important in establishing trust between participants and researchers, and may reduce

For peer review only

the perception of risk and encourage study enrolment (11,28). While this may encourage participation in research, it is important that blind faith in research staff is not influencing a participant's decision-making, nor that participants feel a pressure to conform with the desires of healthcare staff (35).

Altruism

A major influence in favour of enrolling described by pregnant women was altruism. They were encouraged to enrol partly due to potential benefit to their own baby, but also to help other pregnant women, and progress the science-base and care of pregnant women. Altruism driving enrolment has been well established and should be integral to the understanding clinicians have when approaching and enrolling pregnant women in research (33,39).

Strengths and limitations

The study interviewed 12 pregnant women to assess their views on a hypothetical interventional research study. Although the sample size was relatively small, the semi-structured interview format allowed for rich data to be gleaned from each interview and provided detailed themes which were examined. Interviews were conducted with the group of interest, engaging individuals for whom pregnancy-related research is most important. Discussions were further aided by the capacity participants had gained throughout their pregnancy in weighing decisions and balancing study requirements. A further limitation of the study is that participants had, by chance, relatively high educational attainment and so their views may not be representative of the broader population.

Conclusion

For peer review only

Pregnant women were keen to be involved in research and were largely driven by altruism. Risk was a major consideration for research participants, and their perceptions were influenced by their personal experience. Therapeutic interventions in pregnant women have fallen behind their non-pregnant counterparts following prolonged exclusion from research, which affects treatment options available to this population. Progress has been made in recent years towards inclusion of pregnant women, but more remains to be done to overcome inequality in research inclusion (see Fig. 1).

Recommendations for research

1. Redress the recruitment of pregnant women in research. Recent examples have shown that conducting research in pregnancy can be made safe and transparent. Furthermore, our study demonstrates that pregnant women despite their initial reservations, are keen to engage with research studies.
2. Ensure that research is acceptable and feasible to pregnant women. Pregnant women should be engaged early in the design of research, through Patient and Public Involvement work, and detailed discussions held about study requirements, particularly the utility and scientific justification of each of these.
3. Our data suggest that the method of intervention administration matters greatly to participants. Researchers should consider whether alternative methods of administration may better engage pregnant women in research.
4. Recognise that the exclusion of pregnant women from research has a demonstrable impact on their medical care, and may in fact pose greater risk to them than study enrolment.

Fig. 1. Recommendations for research, four recommendations for how to improve equity of research for pregnant women.

For peer review only

Word Count

4214

Declarations

Ethics approval and consent to participate:

This study was granted ethical approval by Health Research Authority (HRA) London – City & East Research Ethics Committee (Reference: 19/LO/0356). All methods were conducted in accordance with the relevant guidelines and approved study documents. Informed consent was gained from all participants enrolled in this study.

Consent for publication:

Not applicable.

Availability of data and materials:

The data generated and analysed during the current study are not publicly available because ethical approval for their distribution has not been granted, nor have participants consented to it being made public.

Competing interests:

The authors declare that they have no competing interests.

Funding:

RBD was supported by the University of Southampton National Institute of Health Research Academic Clinical Fellowship Scheme. AAT is supported by the Medical Research Council (Clinical Research Training Fellowship MR/V002015/1).

Authors' contributions:

RBD, AAT, RCR, TV and CEJ conceived and designed the study. RBD implemented the study and conducted data analysis. RCR and CEJ provided supervision for the study. TV and CEJ provided supervision of the data interpretation. RBD prepared the manuscript. All authors reviewed, edited and approved the final manuscript.

Acknowledgements:

Not applicable.

For peer review only

Additional files

Additional material 1: Supplementary material 1

File name: Iac-InterviewStudySupp1_20230323.docx

Description: Interview guide. Guide used during semi-structured interviews with participants.

For peer review only

References

1. McCarthy CR. Historical background of clinical trials involving women and minorities. Acad Med. 1994;69(9):695–8.

2. Kachikis A, Eckert LO, Englund JA. Chapter 1 - The history of maternal immunization. In: Leuridan EE, Nunes MC, Jones CE, editors. Maternal Immunization. Academic Press; 2020. p. 3–24.

3. van der Zande ISE, van der Graaf R, Oudijk MA, van Delden JJM. Vulnerability of pregnant women in clinical research. J Med Ethics. 2017;43(10):657–63.

4. Council for International Organizations of Medical Sciences (CIOMS). International Ethical Guidelines for Health-related Research Involving Humans. Geneva: CIOMS publications. 2016. https://cioms.ch/publications/product/international-ethical-guidelines-for-health-related-research-involving-humans/. Accessed 16 May 2022

5. Widnes SF, Schjøtt J. Risk perception regarding drug use in pregnancy. American Journal of Obstetrics & Gynecology. 2017;216(4):375–8.

6. van der Zande ISE, van der Graaf R, Oudijk MA, van Delden JJM. A qualitative study on acceptable levels of risk for pregnant women in clinical research. BMC Med Ethics. 2017;18(1):35.

7. Adam MP, Polifka JE, Friedman J m. Evolving knowledge of the teratogenicity of medications in human pregnancy. American Journal of Medical Genetics Part C: Seminars in Medical Genetics. 2011;157(3):175–82.

8. Gomes MF, de la Fuente-Núñez V, Saxena A, Kuesel AC. Protected to death: systematic exclusion of pregnant women from Ebola virus disease trials. Reprod Health. 2017;14(Suppl 3):172.

9. Taylor MM, Kobeissi L, Kim C, Amin A, Thorson AE, Bellare NB, et al. Inclusion of pregnant women in COVID-19 treatment trials: a review and global call to action. The Lancet Global Health. 2021;9(3):e366–71.

10. Lyerly AD, Little MO, Faden R. The second wave: Toward responsible inclusion of pregnant women in research. Int J Fem Approaches Bioeth. 2008;1(2):5–22.

11. Strömmer S, Lawrence W, Rose T, Vogel C, Watson D, Botell JN, et al. Improving recruitment to clinical trials during pregnancy: a mixed methods investigation. Soc Sci Med. 2018;200:73–82.

12. Marbán-Castro E, Pons-Duran C, García-Otero L, Chen H, Herrera LB, Gil M del M, et al. Acceptability of Clinical Trials on COVID-19 during Pregnancy among Pregnant Women and Healthcare Providers: A Qualitative Study. IJERPH. 2021;18(20):10717.

13. Oude Rengerink K, Logtenberg S, Hooft L, Bossuyt PM, Mol BW. Pregnant womens' concerns when invited to a randomized trial: a qualitative case control study. BMC Pregnancy Childbirth. 2015;15:207.

For peer review only

14. Jamrozik E, Littler K, Bull S, Emerson C, Kang G, Kapulu M, et al. Key criteria for the ethical acceptability of COVID-19 human challenge studies: Report of a WHO Working Group. Vaccine. 2021;39(4):633–40.

15. Bambery B, Selgelid M, Weijer C, Savulescu J, Pollard AJ. Ethical Criteria for Human Challenge Studies in Infectious Diseases. Public Health Ethics. 2016;9(1):92–103.

16. Dorey RB, Theodosiou AA, Read RC, Jones CE. The nonpathogenic commensal Neisseria: friends and foes in infectious disease. Curr Opin Infect Dis. 2019;32(5):490–6.

17. Cartwright KA, Stuart JM, Jones DM, Noah ND. The Stonehouse survey: nasopharyngeal carriage of meningococci and Neisseria lactamica. Epidemiol Infect. 1987;99(3):591–601.

18. Deasy AM, Guccione E, Dale AP, Andrews N, Evans CM, Bennett JS, et al. Nasal Inoculation of the Commensal Neisseria lactamica Inhibits Carriage of Neisseria meningitidis by Young Adults: A Controlled Human Infection Study. Clinical Infectious Diseases. 2015;60(10):1512–20.

19. Dale AP, Theodosiou AA, Laver JR, Roche EF, Hill AR, Gorringe A, et al. Controlled human infection with Neisseria lactamica induces B-cell responses that are cross-reactive with Neisseria meningitidis. The Journal of Immunology. 2020;204(1 Supplement):231.21-231.21.

20. Evans CM, Pratt CB, Matheson M, Vaughan TE, Findlow J, Borrow R, et al. Nasopharyngeal colonization by Neisseria lactamica and induction of protective immunity against Neisseria meningitidis. Clin Infect Dis. 2011;52(1):70–7.

21. Theodosiou AA, Laver JR, Dale AP, Cleary DW, Jones CE, Read RC. Controlled human infection with Neisseria lactamica in late pregnancy to measure horizontal transmission and microbiome changes in mother-neonate pairs: a single-arm interventional pilot study protocol. BMJ Open. 2022;12(5):e056081.

22. Braun V, Clarke V. Using thematic analysis in psychology. Qualitative Research in Psychology. 2006;3(2):77–101.

23. Crozier SR, Robinson SM, Borland SE, Godfrey KM, Cooper C, Inskip HM, et al. Do women change their health behaviours in pregnancy? Findings from the Southampton Women's Survey. Paediatric and Perinatal Epidemiology. 2009;23(5):446–53.

24. Kim C, McEwen LN, Piette JD, Goewey J, Ferrara A, Walker EA. Risk Perception for Diabetes Among Women With Histories of Gestational Diabetes Mellitus. Diabetes Care. 2007;30(9):2281–6.

25. Hammer R, Inglin S. 'I don't think it's risky, but…': pregnant women's risk perceptions of maternal drinking and smoking. Health, Risk & Society. 2014;16(1):22–35.

26. Ortendahl MD PhD M, Näsman Fil lic P. Perception of Smoking-Related Health Consequences among Pregnant and Non-Pregnant Women. The American Journal on Addictions. 2007;16(6):521–7.

27. Robinson M, Pennell CE, McLean NJ, Tearne JE, Oddy WH, Newnham JP. Risk perception in pregnancy: Context, consequences, and clinical implications. European Psychologist. 2015;20(2):120–7.

For peer review only

28. Heaman M, Gupton A, Gregory D. Factors influencing pregnant women's perceptions of risk. MCN Am J Matern Child Nurs. 2004;29(2):111–6.

29. Reynolds WW, Nelson RM. Risk perception and decision processes underlying informed consent to research participation. Social Science & Medicine. 2007;65(10):2105–15.

30. Kost RG, Lee LM, Yessis J, Coller BS, Henderson DK. Assessing Research Participants' Perceptions of their Clinical Research Experiences. Clin Transl Sci. 2011;4(6):403–13.

31. Li M, Chapman GB. Why Do People Like Natural? Instrumental and Ideational Bases for the Naturalness Preference. Journal of Applied Social Psychology. 2012;42(12):2859–78.

32. Dibonaventura M daCosta, Chapman GB. Do decision biases predict bad decisions? Omission bias, naturalness bias, and influenza vaccination. Med Decis Making. 2008;28(4):532–9.

33. Meshaka R, Jeffares S, Sadrudin F, Huisman N, Saravanan P. Why do pregnant women participate in research? A patient participation investigation using Q-Methodology. Health Expect. 2017;20(2):188–97.

34. Montgomery GH, Erblich J, DiLorenzo T, Bovbjerg DH. Family and friends with disease: their impact on perceived risk. Prev Med. 2003;37(3):242–9.

35. Carolan MC. Towards understanding the concept of risk for pregnant women: some nursing and midwifery implications. J Clin Nurs. 2009;18(5):652–8.

36. Krubiner CB, Faden RR, Karron RA, Little MO, Lyerly AD, Abramson JS, et al. Pregnant women & vaccines against emerging epidemic threats: Ethics guidance for preparedness, research, and response. Vaccine. 2021;39(1):85–120.

37. Jasper JD, Goel R, Einarson A, Gallo M, Koren G. Effects of framing on teratogenic risk perception in pregnant women. Lancet. 2001;358(9289):1237–8.

38. Gross D, Fogg L. Clinical trials in the 21st century: the case for participant-centered research. Res Nurs Health. 2001;24(6):530–9.

39. Newburg SM, Holland AE, Pearce LA. Motivation of subjects to participate in a research trial. Applied Nursing Research. 1992;5(2):89–93.

For peer review only

Neisseria lactamica in pregnancy: an interview study - Interview guide

Interview duration: 30 minutes (maximum 45 minutes)

This interview will take around 30 minutes of your time. Please stop me to ask questions if anything is not clear. This is a guide and the interviewer has flexibility to explore unexpected areas raised by the study participants. The interview guide will be reviewed after the first few interviews and small changes may be made to content and order in light of the participants' responses.

The aim of this interview is to understand what your thoughts are about a research study that we might potentially do in the future. There are no right or wrong answers. Your answers will help us to be better prepared for this study in the future.

With your permission, this interview will be audio recorded. All information you provide will be confidential, and any information that could identify you will be removed from all transcripts, and the audio will be deleted at the end of the study.

[*Note: allow prompting to clarify answers or to probe respondents thoughts further*]

Before we begin, do you have any questions or is anything not clear?

[Section 1: Understanding of meningitis]

First of all, we would like to know what you understand about meningitis. Remember there are no right or wrong answers, we are just trying to gauge what people understand.

1. Have you heard of meningitis before? Can you tell me what you understand about by the word "meningitis"?
   [Prompt: Do you worry about your child getting meningitis? Is this something you have thought about before? Do you know anyone how has had meningitis?]

Let me give you some information about meningitis to help with some further questions. Meningitis is an infection of the lining of the brain. It can be caused by bacteria that live in many people's noses and throats. Most of the time these bacteria do not cause any problems, but in some people they can, rarely, cause meningitis, and can make some people very unwell. To prevent meningitis, babies and teenagers are given vaccines. These vaccines protect against many of the bacteria that cause meningitis. However, we do not yet have vaccines that protect against all of the bacteria that cause meningitis. Furthermore, these vaccines are only given from 2 months of age, so they do not protect babies from birth.

We are hoping to carry out a research study in the future to see if we can better protect young babies from meningitis.

[Section 2: Understanding of *Neisseria lactamica* and the microbiome]

Neisseria lactamica is a type of 'good bacteria' that lives in the nose and throat. You might have heard about 'good or friendly bacteria' that can be found in some yoghurts. In healthy people with a normal immune system, 'good' bacteria do not cause any problems. Most people will carry Neisseria lactamica in their noses at some point in their lives, either as young children or as parents of young children.

For peer review only - http://bmjopen.bmj.com/site/about/guidelines.xhtml

We are thinking about doing a study where we would give Neisseria lactamica into the noses of pregnant women.

2. What is your first reaction to hearing this? How does this information make you feel?
3. How likely would you be to take part in a study which gave *Neisseria lactamica* into your nose whilst you were pregnant? How likely would you be to take part, with 1 being 'never' to 10 being 'definitely'? Can you explain why?

As I explained before, Neisseria lactamica is a type of 'good' bacteria that lives in the nose and throat. Having this bacteria in the nose and throat is common in childhood, with around 30% of children carrying it at any one time.

There is evidence that during outbreaks of meningitis, people who already have Neisseria lactamica in their noses are less likely to get meningitis. We have done studies in healthy adults showing that people who are given Neisseria lactamica into their noses were less likely to carry the bacteria that can cause meningitis. This is why we want to investigate Neisseria lactamica, to see whether we can protect people from meningitis, in particular young infants. We would aim to give pregnant women Neisseria lactamica into their noses at the end of pregnancy and see whether Neisseria lactamica is transferred to their babies noses in the few weeks after birth. We want to see if this changes the types of bacteria that babies carry in their noses, from less healthy types to more healthy types.

The human body is made up of more bacterial cells than human cells, the vast majority of which will never cause any problem. If someone has a severely weakened immune system, then any of these bacteria could theoretically cause infection, including Neisseria lactamica. However, there isn't any evidence that Neisseria lactamica causes infection in healthy adults.

[Section 3: Acceptability the *Neisseria lactamica* study]

4. After hearing more information, how likely is it that you would you take part in this study now? Can you rate how likely you would be to take part, with 1 being 'never' and 10 being definitely. Can you explain why?
   [Prompt: what concerns do you have? For example: Safety? If 10 people had already been given the bug would you accept it? What about if 1000 people or 10,000 people had?]

In the medical literature, there are three cases described which show Neisseria lactamica causing an infection in healthy children. These cases were reported between 1976 and 1978, and there have been no reported cases internationally since then.

5. Does this information change your mind about taking part in a research study involving *Neisseria lactamica*? Does this make the study seem more risky? What would reassure you?
6. What do you deem to be the most important risks?
   [Prompt: risk to you, risk to your baby?]
7. What other information would you require before considering participating? Which parts of the information you have heard, are most important to you, and why?
   [Prompt: Have we provided all the information that you would like to know?]
8. Having spoken about your concerns in the question before, if we provided all of this information, how likely are you now to participate in the study: 1 (never) -10 (definitely)?

For peer review only - http://bmjopen.bmj.com/site/about/guidelines.xhtml

[Prompt: Which information has helped you make up your mind?]

9. What information would most encourage you to participate in this study?
10. What information would put you off participating in this study?
11. We may not give *Neisseria lactamica* into the noses participants who are found already have it in their nose. If you were found to have low levels of *Neisseria lactamica* in your nose how would feel about:
    a. Being given the *Neisseria lactamica* spray into your nose to increase the amount that is present there?
    b. Not being given anything, but being followed-up as described above?

Study design of *Neisseria lactamica* study

12. How regularly would you deem it acceptable to come to clinic for this study during pregnancy (1-2 times, 3-4 times, 5-6 times, monthly)?
    [Prompt: would you be willing to be seen more regularly if we came to your home? How else could we facilitate participation in follow-up?]
13. How often would you a be prepared to attend follow-up with your baby?
    [Prompt: once at birth, two/three/ four/six times in the first year of life. How could we facilitate participation in follow-up?]
14. In addition to any sample collected as part of routine care, what samples would be acceptable to collect from:
    a. You - blood, throat swabs, nose wash, breast milk
    b. Infant - blood, nose swabs, throat swabs

Conclusion

15. Do you think this study is ethical?
    [Prompt: Do you think this study is acceptable to you? if yes, why? If no, what would need to change, and what other information would you need?]
16. Do you think this study should be done?
    Prompt: if yes, why? If no, why?
17. Is there anything else you would like to share or ask?

Finally, some questions about you:

18. How many weeks pregnant are you?
19. Have you had any children before? *(if no, move to question 20)*
20. How old are your children?
21. Have you participated in research before? During pregnancy?
22. Which ethnic group do you feel you belong to? [Prompt: use codes]

Thank you

For peer review only - http://bmjopen.bmj.com/site/about/guidelines.xhtml

Qualitative interview study exploring the perspectives of pregnant women on participating in controlled human infection research in the UK

Journal:	*BMJ Open*
Manuscript ID	bmjopen-2023-073992.R1
Article Type:	Original research
Date Submitted by the Author:	22-Nov-2023
Complete List of Authors:	Dorey, Robert; University of Southampton Faculty of Medicine; University Hospital Southampton NHS Foundation Trust, NIHR Southampton Clinical Research Facility and NIHR Southampton Biomedical Research Centre<br>Theodosiou, Anastasia; University of Southampton, Clinical and Experimental Sciences; University Hospital Southampton NHS Foundation Trust, NIHR Southampton Clinical Research Facility and NIHR Southampton Biomedical Research Centre<br>Read, Robert; University of Southampton Faculty of Medicine; University Hospital Southampton NHS Foundation Trust, NIHR Southampton Clinical Research Facility and NIHR Southampton Biomedical Research Centre<br>Vandrevala, T; Kingston University and St George's University of London, Centre for Applied Health and Social Care Research<br>Jones, Christine; University of Southampton Faculty of Medicine; University Hospital Southampton NHS Foundation Trust, NIHR Southampton Clinical Research Facility and NIHR Southampton Biomedical Research Centre
**Primary Subject Heading**:	Qualitative research
Secondary Subject Heading:	Obstetrics and gynaecology, Paediatrics, Patient-centred medicine, Reproductive medicine
Keywords:	QUALITATIVE RESEARCH, Maternal medicine < OBSTETRICS, Paediatric infectious disease & immunisation < PAEDIATRICS, NEONATOLOGY, OBSTETRICS, MICROBIOLOGY

I, the Submitting Author has the right to grant and does grant on behalf of all authors of the Work (as defined in the below author licence), an exclusive licence and/or a non-exclusive licence for contributions from authors who are: i) UK Crown employees; ii) where BMJ has agreed a CC-BY licence shall apply, and/or iii) in accordance with the terms applicable for US Federal Government officers or employees acting as part of their official duties; on a worldwide, perpetual, irrevocable, royalty-free basis to BMJ Publishing Group Ltd ("BMJ") its licensees and where the relevant Journal is co-owned by BMJ to the co-owners of the Journal, to publish the Work in this journal and any other BMJ products and to exploit all rights, as set out in our licence.

The Submitting Author accepts and understands that any supply made under these terms is made by BMJ to the Submitting Author unless you are acting as an employee on behalf of your employer or a postgraduate student of an affiliated institution which is paying any applicable article publishing charge ("APC") for Open Access articles. Where the Submitting Author wishes to make the Work available on an Open Access basis (and intends to pay the relevant APC), the terms of reuse of such Open Access shall be governed by a Creative Commons licence – details of these licences and which Creative Commons licence will apply to this Work are set out in our licence referred to above.

Other than as permitted in any relevant BMJ Author's Self Archiving Policies, I confirm this Work has not been accepted for publication elsewhere, is not being considered for publication elsewhere and does not duplicate material already published. I confirm all authors consent to publication of this Work and authorise the granting of this licence.

For peer review only

Title

Qualitative interview study exploring the perspectives of pregnant women on participating in controlled human infection research in the UK

Robert B Dorey, Anastasia A Theodosiou, Robert C Read, Tushna Vandrevala, Christine E Jones

Affiliations:

Robert B Dorey BMBS, MSc, BMedSci, MRCPCH: Faculty of Medicine, University of Southampton; and NIHR Southampton Clinical Research Facility and NIHR Southampton Biomedical Research Centre, University Hospital Southampton NHS Foundation Trust, Southampton, United Kingdom

Anastasia A Theodosiou MA, MPhil, MBBChir, MRCP: Clinical and Experimental Sciences; and NIHR Southampton Clinical Research Facility and NIHR Southampton Biomedical Research Centre, University Hospital Southampton NHS Foundation Trust, Southampton, United Kingdom

Robert C Read MD, FRCP, MBChB, BMedSci, FIDSA, FESCMID: Faculty of Medicine and Institute for Life Sciences, University of Southampton; and NIHR Southampton Clinical Research Facility and NIHR Southampton Biomedical Research Centre, University Hospital Southampton NHS Foundation Trust, Southampton, United Kingdom

Tushna Vandrevala PhD, MSc, BA, CPsychol: Centre for Applied Health and Social Care Research, Faculty of Health, Social Care and Education, Kingston University and St George's University of London, London, United Kingdom

Christine E Jones BMedSci, BMBS, PgCertHBE, FRCPCH, FHEA, PhD: Faculty of Medicine and Institute for Life Sciences, University of Southampton; and NIHR Southampton Clinical Research Facility and NIHR Southampton Biomedical Research Centre, University Hospital Southampton NHS Foundation Trust, Southampton, United Kingdom

Corresponding author:

Robert B Dorey: Rob.Dorey@ggc.scot.nhs.uk

For peer review only

Abstract

Introduction:

Pregnant women have been historically excluded from interventional research. While recent efforts have been made to improve their involvement, there remains a disparity in the evidence base for treatments available to pregnant women compared with the non-pregnant population. A significant barrier to the enrolment of pregnant women within research is risk perception and a poor understanding of decision-making in this population.

Objective:

Assess the risk perception and influences on decision-making in pregnant women, when considering whether to enrol in a hypothetical interventional research study.

Design:

Semi-structured interviews were undertaken, and thematic analysis was undertaken of participant responses.

Participants:

Twelve pregnant women were enrolled from an antenatal outpatient clinic.

Results:

Participants were unanimously positive about enrolling in the proposed hypothetical interventional study. Risk perception was influenced by potential risks to their fetus, and their previous experiences of healthcare and research. Participants found the uncertainty in quantifying risk for new research interventions challenging. They were motivated to enrol in research by altruism and found less invasive research interventions more tolerable.

For peer review only

Conclusion:

It is vital to understand how pregnant women balance the perceived risks and benefits of interventional research. This may help clinicians and scientists better communicate risk to pregnant women,and address the ongoing underrepresentation of pregnant women in interventional research.

Strengths and Limitations

- Relatively small sample size, although semi-structured interview format allowed for rich data to be gleaned and provided detailed themes which were examined.
- Conducted with the group of interest, engaging individuals for whom pregnancy-related research is most important.
- Participants had gained experience throughout pregnancy in weighing decisions and balancing medical decisions, allowing for excellent engagement in discussion about study enrolment.
- Participants had relatively high educational attainment and so may not be representative of the broader population.

Key words

Pregnancy, neonate, human challenge, qualitative, thematic analysis, interview, risk perception, decision-making, research participation

Background

Interventional research in pregnant women

Interventional research in pregnancy has remained contentious since the formalisation of human research ethics in the mid-20th Century [1,2]. Although neither the Nuremberg Code (1947) nor the

For peer review only

World Medical Association's Declaration of Helsinki (1964) explicitly referred to pregnant women, their participation in interventional research was restricted following a number of highly publicised cases of harm caused by medical interventions in pregnancy [1]. For instance, the use of thalidomide in pregnancy resulted in many thousands of congenital anomalies and infant deaths in the 1960s, while antenatal use of diethylstilbestrol was recognised as a cause of cancer in women and their female offspring in the 1970s [1–3]. Due to perceptions of greater susceptibility to harm, pregnant women were increasingly excluded from interventional research, and even reclassified as a "vulnerable population" by the U.S. Department of Health and Human Services in 1975 [1,2].

The 1980s saw a shift towards greater inclusion of pregnant women in research studies. With dramatically increasing medical costs in the United States, research offered the public the possibility of subsidised medical care [1]. Furthermore, before anti-retroviral therapies were made widely available for the treatment of HIV, clinical research participation was viewed as a means of accessing medication for an otherwise fatal infection. As the incidence of HIV in women increased, there was a rise in public opinion that the exclusion of women from HIV research studies was discriminatory. This context increased political pressure to expand inclusion of pregnant women in interventional research [1]. Subsequently, in 1985, the U.S. Public Health Service Task Force publicly recognised a lack of data specific to women's health, and Congress established a Department of Women's Health at the National Institutes for Health through the passage of the Women's Health Equality Act (1990), thus mandating research in this area [2]. This new sentiment was further enforced by international guidance from Council for International Organizations of Medical Sciences (CIOMS) in 1993 characterising the exclusion of pregnant women from research as "unjust" [4].

Despite this progress, concerns remain among clinicians and researchers that interventional studies may pose a risk of serious harm to the mother and fetus. There is evidence that clinicians over-estimate the risk of teratogenicity caused by medications during pregnancy [5]. Further, ethics

For peer review only

committees appear more likely to perceive interventional studies in pregnancy as high risk, due to difficulties in quantifying or excluding risk of harm to the fetus [6]. Indeed, a study involving interviews with ethics committee members found that many members would not question the exclusion of pregnant women from research, and felt it was easier to exclude them from interventional research and opt for observational studies instead [6]. These concerns may contribute to ongoing exclusion of pregnant women from interventional research. Indeed, of 172 medications approved by the U.S. Food and Drug Administration between 2000 and 2010, 97.7% has "undetermined" teratogenicity risk, while safety data relating to pregnancy was reported as "none" for 73.3% [7]. Even within the last decade, pregnant women were excluded from trials of Ebola treatment and vaccines during the outbreak of 2013-2016 [8], and from up to 80% of relevant interventional studies during the first year of the SARS-CoV2 pandemic in 2020, often without specific pregnancy-related safety concerns regarding the investigational medicinal product [9]. In the absence of interventional research, many treatment decisions in pregnancy therefore rely on extrapolating from research in non-pregnant individuals and post-marketing data, which may be associated with use of outdated or inferior treatments, and even worse clinical outcomes [3,10].

Even when pregnant women are eligible to participate in interventional research, they may be reluctant to enrol. Concerns amongst pregnant women include the harm that enrolling in interventional studies may cause their fetus, and a lack of knowledge about the investigational product [11,12]. Furthermore, interview studies have highlighted a perception that enrolling in research may cause participants stress, something they wish to minimise during pregnancy [12,13]. This may be a legacy of the exclusion from research that pregnant women have experienced, and continues to contribute to the perception that all research in pregnancy is potentially harmful, regardless of the relative risks associated with a particular intervention [11]. An improved understanding of how pregnant women balance risks and make decisions regarding research

For peer review only

involvement may allow better engagement of pregnant women by research staff and allow individuals to overcome perceived barriers.

Controlled human infection research in pregnancy

Controlled human infection (also known as human challenge) is a type of interventional research that involves exposing participants to a defined number of live micro-organisms to investigate host response, colonisation, and infection kinetics, and even the effect of antimicrobial products or vaccines. Much like research in pregnancy, this type of research presents unique ethical and practical considerations over and above those shared by all interventional research [14,15]. These include balancing the risk to the participant and their contacts by exposure to a potentially harmful organism, against the direct benefits to the participant and potential future health benefits for society.

Prior to the study described below, ethically-approved controlled human infection research had not been conducted in pregnant women. A growing body of evidence has highlighted the importance of maternal to infant microbe transmission in shaping the neonatal upper respiratory microbiome and long-term adverse health outcomes such as respiratory infections and asthma [16,17]. These findings have led to calls for interventional research in pregnancy and early life, to further investigate the role of maternal-infant microbe transmission in child health, and to explore new avenues for preventing and treating childhood infections, such as mucosal inoculation with beneficial microbes [18]. Thus, the Lactamica 9 study was proposed as a human challenge study in pregnancy, using *Neisseria lactamica* nasal inoculation in pregnant women to investigate for mother-to-infant upper respiratory commensal transmission and impact on infant microbiome and immune development [16].

Neisseria lactamica is a type of harmless commensal bacteria that has been well-characterised in human challenge research involving healthy non-pregnant adults. It is found in the upper respiratory

For peer review only

tract of over 40% of 1-2 year old infants, although it is present in fewer than 10% of adults and neonates [19,20]. *N. lactamica* exhibits an inverse relationship with *N. meningitidis* colonisation and invasive disease, and in controlled human infection studies, it has been shown to displace *N. meningitidis* from the upper respiratory tract by inducing cross-reactive humoral and cellular immunity [21–23]. In the immunocompetent host, *N. lactamica* does not cause clinical disease, and has been safely administered intranasally to over 400 non-pregnant adults in human challenge studies. Given the novelty of the proposed Lactamica 9 study, and the unique ethical and practical considerations of performing human challenge research in pregnant women, a preparatory study was conducted using semi-structured interviews with pregnant women. These interviews aimed to gain insights into pregnant women's perception of risk and decision-making when considering hypothetical participation in a controlled human infection study. The results of the interview study are presented below, and were used to inform the design and ultimate execution of the Lactamica 9 study. In doing so, we aimed to identify and address barriers to research involvement, facilitate better engagement with pregnant women, and ultimately improve equitable participation in this population.

For peer review only

Methods

Women were approached in an antenatal clinic at a large teaching hospital in the UK, between March 2019 and February 2020. All women attending an antenatal clinic were approached whilst in the waiting room, and their initial interest was determined. Those who expressed interest were given more information about the study, and, if they wished to proceed, informed consent was gained. Women were eligible if they were aged over 18 years and receiving antenatal care at the site; and were excluded if they were not able to understand written and spoken English. The interview study

was approved by Health Research Authority (HRA) London – City & East Research Ethics Committee (Reference: 19/LO/0356).

Face-to-face, semi-structured interviews were carried out with 12 pregnant women, lasting between 30 and 60 minutes each, and all interviews were audio recorded. The interview guide consisted of open-ended questions and prompts, and follow-up questions were asked to further explore themes raised by participants (see Supplementary material 1). The interview asked participants to reflect on their hypothetical participation in a proposed study and explored participants' understanding of invasive meningococcal disease, perception of risk, and acceptability of different study designs, procedures and interventions. In particular, participants were asked to consider live bacterial inoculation using nasal drops, and collection of upper respiratory (nose, throat and mouth) swabs, breast milk and blood from themself and their newborn baby. Following the interviews, recordings were transcribed verbatim and transcripts were anonymised. No further interviews were conducted once data saturation (the point at which each new interview produces only previously discovered data) was reached.

Data was managed and analysed using NVivo (version 1.6.1). Data analysis was performed in five stages as described by Braun and Clarke [24]: i) familiarisation – researchers were familiarised with data prior to analysis so that broad content was understood; ii) code generation – transcripts were summarised using codes, where each code described a segment of data using a key analytic idea; iii) searching for themes – following code generation, researchers combined different codes under broader generalised patterns, called themes; which described commonalities between codes; iv) reviewing themes – once identified, codes were better defined using central organising concepts, and these themes were reviewed to ensure that they accurately represented the data, particularly as it related to the research theme; v) defining and naming themes. Codes and themes were cross-checked, discussed, and reviewed with two other researchers (CJ, TV) to ensure accurate representation of

For peer review only

participant responses. Themes were analysed in relation to the research question, and data interpreted in this context.

Patient and Public Involvement

Prior to this study, pregnant women were approached while attending an antenatal parent evening at a maternity hospital. Pregnant women and their partners who were interested were engaged in an informal discussion regarding the proposed study. The study design was explained, including interview content, duration and study aims. Opinions were then gathered regarding phrasing of questions, possible locations of recruitment, and strategies and factors that would encourage or dissuade engagement. Eight couples were involved in this process, and this information informed the design of the presented study in a number of ways. Firstly, participants were recruited at an antenatal clinic in a maternity hospital as it was felt that this would reduce disruption for participants enrolling in the study. Secondly, the language used in interviews was changed so that it might be less alarming to participants; for example, the scientific term "bacteria" was preferrable to colloquial term "bugs". Finally, it was felt that an interview duration of 30 to 60 minutes would be acceptable, especially given intervals of waiting during the antenatal clinic from which study participants were recruited."

<u>Results</u>

Twelve pregnant women were interviewed regarding their perceptions of risk and their decision-making about hypothetical participation in a controlled human infection study in pregnancy. Participants were women of childbearing age (25 to 40 years) with a range of educational attainment (see Table 1). Broadly, participants were white British (11/12), and seven participants did not have any children. The majority of participants (11/12) would, in principle, agree to participate in the interventional study proposed.

For peer review only

Table 1.

Characteristic		Participant number (%)
Marital status	Single	7 (58)
	Married	5 (42)
Gestation	First trimester	1 (8)
	Second trimester	6 (50)
	Third trimester	5 (42)
Gravida	Gravida 1	6 (50)
	Gravida 2	5 (42)
	Gravida 4	1 (8)
Parity	Parity 0	7 (58)
	Parity 1	4 (33)
	Parity 3	1 (8)
Ethnicity	White British	11 (92)
	Any other Asian background	1 (8)
Time in the UK	Since birth	11 (92)
	15 years or more	1 (8)
Disability or long-term health condition	No	9 (75)
	Yes	3 (25)
Highest qualification	GCSE/BTEC or equivalent	3 (25)
	AS / A-Levels or Scottish higher or equivalent	3 (25)
	BSc / BA or equivalent	5 (42)
	PhD or equivalent	1 (8)
Previous participation in research	No	11 (92)
	Yes	1 (8)

For peer review only

Overall, our data suggests that pregnant women have a heightened perception of risk, compared with when they were not pregnant. They balanced the risks and benefits of enrolling in an interventional study in the context of their individual experiences, and were motivated by the potential for developing new treatments. The acceptability of a research study was closely linked to the interventions that would be undertaken, and participants perceived uncertainty about risk to be equivalent to a high risk of harm (see Table 2 for themes and subthemes).

Table 2.

**Theme**	**Subtheme**
Heightened perception of risk during pregnancy	Gestational age changes risk perception
	Babies cannot consent
Balancing risks and benefits by pregnant women	Prior understanding and experiences influence decision-making
	Conflation of uncertainty and risk
Factors which encourage participation in research	Altruism
	Research processes are reassuring
	Acceptability of study procedures

Theme 1: Heightened perception of risk during pregnancy

Participants were concerned about the risks associated with participating in research during pregnancy. This concern was attributed to two main factors: concern for the health of their unborn baby, and that they themselves may be at higher risk by virtue of being pregnant. Women expressed

For peer review only

a heightened perception of risk while pregnant, with even a small amount of risk to their baby being intolerable, although a degree of risk to themselves was felt to be more acceptable.

> *"My first and foremost concern would obviously be the safety to my baby. And then, secondary to that, myself"* (Participant 9, 26 weeks gestation, second pregnancy)

> *"With my baby, even a small amount of risk is not tolerable"* (Participant 1, 28 weeks gestation, first pregnancy)

Subtheme 1.1: Gestational age changes risk perception

Women's perception of risk was related to the gestational timing of the proposed research study, although this effect varied between individuals. Some participants reported that they would avoid participating in research in early gestation, as they perceived greater risk of complications and felt more uncertain about their pregnancy at this time. Conversely, other participants reported greater reservations about participating later in pregnancy, as they expressed more attachment to their fetus, greater confidence that the pregnancy was going to progress to birth, and that their fetus was more like an individual (in part because they could feel it move).

> *"I suppose at 36 weeks … If anything were to go wrong, 36 weeks is a very good threshold that they can convert to be induced I suppose. But yeah, that has made me think more about participating"* (Participant 7, 26 weeks gestation, first pregnancy)

> *"If I knew there was going to be a high risk to harming the baby, then I probably wouldn't because if you're doing it later on in pregnancy. Obviously that baby is already a part of you.*

For peer review only

You can feel it move, you can feel it kick and you get used to its routine and stuff like that." (Participant 4, 34 weeks gestation, first pregnancy)

Subtheme 1.2: Babies cannot consent

For some participants, the inability of the fetus to consent to research participation was paramount when considering hypothetical involvement in the proposed study. Some suggested that, since the research could theoretically cause long-term complications, they did not feel it was acceptable to consent on the unborn child's behalf. Furthermore, the baby's inability to consent would increase their own feelings of guilt if complications or adverse outcomes arose.

"I go into it knowing about it, my baby doesn't. So, I would be more worried about what it would do to my baby." (Participant 2, 12 weeks gestation, first pregnancy)

Theme 2: Balancing risks and benefits by pregnant women

For most participants, their views on hypothetical participation in the proposed human challenge study were based on a balance of perceived benefits of enrolment against potential risks and adverse events. Being pregnant brought with it, an even greater responsibility to carefully balance these risks and benefits.

Subtheme 2.1: Prior understanding and experiences influence decision-making

Previous experiences of invasive meningococcal disease influenced how they viewed their hypothetical involvement in such research. Women with personal experience, or who knew someone

For peer review only

affected by the condition being investigated, gave greater weight to the importance and potential benefits of the study and were therefore generally supportive of study participation.

> *"It wasn't on my radar, until a friend of mine has recently had a scare with meningitis. Her little girl had a rash that wouldn't disappear, so they took her up to hospital. But it seemed it wasn't meningitis. Then, they kind of, it does play on your mind a bit."* (Participant 7, 26 weeks gestation, first pregnancy)

Previous experience of research or medical care also weighed greatly on participants' decision-making. More specifically, traumatic experiences, particularly related to medical procedures, increased perceived risk. For example, a previous child having a difficult experience with venesection would make a study requiring neonatal blood tests appear more risky or unacceptable.

> *"I had to take my daughter quite young for a blood test. And I remember her, she was really distressed"* (Participant 10, 33 weeks gestation, second pregnancy)

For some participants, risk perceptions about study participation during pregnancy appeared entrenched and binary; either research would provide significant benefits warranting participation, or posed significant risks precluding participation. For these individuals, perspectives on risks and benefits of research did not change during the interview, despite information and safety data provided by the research team. Further, their stance did not appear specific to this particular study, but rather applied more broadly to research participation regardless of proposed interventions or procedures. In contrast, other individuals who were undecided about hypothetical study participation at the start of the interview appeared to favour participation in the hypothetical study, following provision of further information by the interviewer.

For peer review only

"Yeah, but if you already knew, I take it as if you already know information anyway and you have an opinion about it, you're going to go for it or against it. Either way. Your mind is already made up … I think it would just reassure the ones that maybe are going to, maybe people sitting on the fence might be swayed a bit more, but the "no"s definitely wouldn't be, I don't think it would affect, I don't think it would change their mind." (Participant 6, 27 weeks gestation, first pregnancy)

Subtheme 2.2: Conflation of uncertainty and risk

Participants were wary of the uncertainties involved in enrolling in an interventional study, especially one that has not previously been undertaken in pregnancy. They expressed difficulty in balancing proposed benefits with the potential for unknown complications, and considered the impact on their fetus more than themselves. They perceived uncertainty about potential harm in much the same way as they would known risks. Indeed, they struggled to rationalise participation when research staff could not unequivocally guarantee no risk associated with study participation.

"Because, still, you can't guarantee it won't cause any problems" (Participant 8, 34 weeks gestation, second pregnancy)

Specific concerns were that the intervention itself could cause harm. While participants recognised that undertaking research is required to improve care for pregnant women and neonates, some felt that participation in interventional research posed greater risk to them personally than the prospect of insufficient research. This perception was heightened as they were told there may be no direct benefits to the mother or neonate of participating.

For peer review only

"I don't know. I really don't know. I think that sitting on the fence. You would never want to see your baby ill, but you also wouldn't want to put your baby in jeopardy if this is something that didn't work... That's just the way I think about it." (Participant 1, 28 weeks gestation, first pregnancy)

Theme 3: Factors which encourage participation in research

Despite the perceived uncertainties and risks, the participants were nearly unanimous in support of hypothetical participation in such a human challenge research study. Their support was mainly related to specific features of the proposed study, as well as a desire to further the care of pregnant women and neonates in general.

Subtheme 3.1: Altruism

A significant motivator for engaging with research was altruism. There was a perception that engaging with research as a pregnant woman could improve the care available to pregnant women and neonates, providing benefit to both themselves and others. Participants identified with other pregnant women and new parents, and felt motivated to improve research to reduce the chance of other families bearing the burden of disease and illness.

"I guess helping in medical research as well, meningitis can be fatal. And if you can stop another child and another family having to go through that, that would be something nice to do." (Participant 4, 34 weeks gestation, first pregnancy)

"I just think, why wouldn't you try and help if you could?" (Participant 6, 27 weeks gestation, first pregnancy)

For peer review only

Subtheme 3.2: Research processes are reassuring

Participants were reassured by the safety procedures in place regulating clinical trial. They felt confident that inclusion and exclusion criteria meant they would not be enrolled if it were inappropriate or unsafe. Further, they took comfort in knowing that they, and their baby, would be regularly reviewed by a clinician; and that if adverse events arose (affecting other participants or themselves), they would be investigated and managed according to study procedures.

> *"And knowing all of these checks, there are going to be hurdles you have to get through to take part, is good."* (Participant 2, 12 weeks gestation, first pregnancy)

> *"I would feel happier about it now knowing there are contingencies in place."* (Participant 2, 12 weeks gestation, first pregnancy)

Subtheme 3.3: Acceptability of study procedures

The proposed study intervention and sampling procedures influenced perceived acceptability. When presented with a range of sampling procedures, participants expressed greater willingness to participate if less invasive procedures were proposed, and even appeared to perceive reduced risk regarding the study as a whole (despite not altering the proposed study intervention). In particular, participants felt that research involving needles was less acceptable than those without needles, and they described a preference for heel prick sampling over venesection to acquire neonatal blood. Moreover, the proposed intervention (inoculation with live *N. lactamica*) was considered more natural, more acceptable and less risky, if administered via nasal drops than via intramuscular injection.

For peer review only

"That would be more acceptable to me, just because, it's (heel prick blood test) quick, it's simple, and I think it's not as, I think it's not as dramatic as a baby, for a baby, but yeah, it's easier" (Participant 6, 27 weeks gestation, first pregnancy)

"I think that's fine, it's not that invasive, to you or the baby, it's just a spray. So that part wouldn't really put me off." (Participant 3, 26 weeks gestation, second pregnancy)

"Well, I guess the natural version will always be more preferable" (Participant 6, 27 weeks gestation, first pregnancy)

For peer review only

Discussion

Understanding and communicating risk is complex, and most research to date on risk perception in pregnant women has focused on lifestyle measures rather than participation in interventional research [25–28]. This interview-based study identified differences in participants' conceptualisation of risk, although most participants cited uncertainty, invasiveness, or personal experience as contributors to their perception of risk. Further, many participants perceived risk as binary, rather than graduated based on exposure and study-specific considerations.

Uncertainty affects perception of risk

The challenge of understanding and communicating risk may be compounded by pregnant women's apparent conflation of risk and uncertainty. In the context of research participation, risk refers to the probability of harm following an intervention, while uncertainty relates to unknown or unexpected

consequences (which may or may not be harmful). Participants often equated uncertainty with harm, viewing it as a major barrier to hypothetical participation in interventional research. This finding has been reported in past literature, with pregnant women reporting a preference to let matters take their "natural course" [12,13]. Thus, they distinguish between an adverse outcome arising through inaction (i.e. choosing not to participate in research) and through an action that subsequently causes harm (i.e. agreeing to a research intervention) [13].

This conflation of risk and uncertainty may be a reflection of modern obstetrics, where, due to advances in antenatal care, so much is known about a pregnancy that uncertainty is marginalised and so the unknown becomes perceived as a risk [29,30]. This misconception is pervasive and affects not only how pregnant women perceive themselves, but also how healthcare staff and researchers see them, and may thus contribute to the ongoing exclusion of pregnant women from interventional research.

Invasiveness of intervention affects perception of risk

The nature of research interventions and procedures influence how risk is perceived by potential participants. Based on our small sample of pregnant women, participants expressed a preference for nasal compared with intramuscular inoculation, and for heel-prick sampling over infant venesection. Past research has reported reduced likelihood of study enrolment if venesection is required in children [31], while painful interventions in general are associated with negative participant experience while enrolled in research studies [32]. The reasons for aversion to needles are likely complex; however, in our participants, procedures involving needles were perceived to be more invasive, and therefore less acceptable and more risky. Furthermore, our participants felt that the proposed intervention utilised a natural process (neonatal colonisation with maternal bacterial flora), and they found this more acceptable than other interventions they were asked to reflect on, such as injectable vaccines. The

For peer review only

preference of society for natural products is well described [33,34], and it may be that using non-needle-based interventions may allay concerns and alter risk perception of interventional research in pregnancy.

Prior health-related experience affects perception of risk

The interviews demonstrated that how participants perceive risk and weigh decisions is greatly influenced by their prior experiences. The effect of previous experience on risk perception has previously been described, and is maintained even if there is no effect on absolute risk; for example, in genetic diseases affecting non-familial relations [35,36]. Research participants who decline to be involved in studies have reported different levels of perceived risk to those who go on to enrol in research, and the information presented does not appear to change the likelihood of enrolment [11]. It appears that prior life experience is important in risk perception and decision-making, and even before participants are approached, they may be pre-determined to either enrol or not based on these experiences.

Previous studies have demonstrated that, while clinicians typically present risk numerically (in terms of incidence or odds), pregnant women perceive risk in a more individualised way, relying more on personal experience than epidemiology [37].

Making research and treatment more equitable

There has been a shift away from protectionist policies in recent years, and inclusion of pregnant women within research has improved [2]. However, there remains a wide disparity between engagement of pregnant women compared with non-pregnant participants in interventional research [8,9]. Even recent calls “to protect [pregnant] groups through research, not just from research” [38]

For peer review only

retain vestiges of protectionist sentiment: the focus remains on researchers and governing bodies assessing risk on behalf of pregnant women, rather than empowering women to assess risk and decide whether to enrol.

Communicating risk

We have highlighted, as have others [37,39], that understanding risk is challenging for all involved in research. Research needs be made more accessible to pregnant women by improving the appreciation of the differences in risk conceptualisation between researchers and pregnant women, and improved understanding that balancing risk may depend on personal previous experience.

Patient and Public Involvement

A major barrier for pregnant women considering whether to enrol in research are logistical difficulties [11,40]. Research protocols can be too demanding for participants, especially for those in full-time employment or with caring responsibilities. Patient-focussed research can be improved by involving patients early in study design through PPI (Patient and Public Involvement) groups. In addition to addressing logistical issues, this would highlight important areas of concerns that we have found in this study regarding study procedures and allow for considered amendments to protocols; excluding procedures that do not add sufficient value in answering the research question.

Reassured by healthcare professionals

Pregnant women may be reassured by the stringent requirements placed upon researchers by governing bodies. Participants were encouraged that researchers thought the study was of value. Confidence in research staff is important in establishing trust between participants and researchers,

For peer review only

and may reduce the perception of risk and encourage study enrolment [11,30]. While this may encourage participation in research, it is important that blind faith in research staff is not influencing a participant's decision-making, nor that participants feel a pressure to conform with the desires of healthcare staff [37].

Altruism

A major influence in favour of enrolling described by pregnant women in this study was altruism. They were encouraged to enrol partly due to potential benefit to their own baby, but also to help other pregnant women, and progress the science-base and care of pregnant women. Altruism driving enrolment has been well established and should be integral to the understanding clinicians have when approaching and enrolling pregnant women in research [35,41].

Study strengths, limitations and applications

The study included interviews with twelve pregnant women to assess their views on hypothetical participation in a controlled human infection study. The results of these interviews meaningfully influenced the design and ultimate execution of the Lactamica 9 study, including offering study visits in the participants' homes, limiting study follow-up to four months post-partum, and collecting umbilical cord blood rather than infant venous blood at birth [16].

Although the sample size was relatively small, the semi-structured interview format allowed for rich data to be gleaned from each interview and provided detailed themes which were examined. Interviews were conducted with the group of interest, engaging individuals for whom pregnancy-related research is most important. Discussions were further aided by the capacity participants had gained throughout their pregnancy in weighing decisions and balancing study requirements.

For peer review only

It is noteworthy that interviewees had relatively high educational attainment, and most were ethnically White British. Perceptions appeared consistent between individuals of different educational attainment, but there may be limitations to the generalisability of these results. However, one of the primary aims of this interview study was to inform the eventual design of the Lactamica 9 controlled human infection study, which recruited participants receiving antenatal care at the same large teaching hospital. Thus, although potentially not generalisable beyond the study population, the sample of interviewees may nonetheless be representative of the Lactamica 9 study's recruitment population.

Conclusion

Pregnant participants were keen to be involved in research and were largely driven by altruism. Risk was a major consideration, and their perceptions were influenced by their personal experience. Therapeutic interventions in pregnant women have fallen behind their non-pregnant counterparts following prolonged exclusion from research, which affects treatment options available to this population. Progress has been made in recent years towards inclusion of pregnant women, but more remains to be done to overcome inequality in research inclusion (see Fig. 1).

For peer review only

Tables and Figures

Table 1. Demographic characteristics of participants.

Table 2. Summary of themes and subthemes present in interviews with pregnant women.

Fig. 1. Recommendations for research. Four recommendations for how to improve equity of research for pregnant women.

For peer review only

Word Count

4299

Declarations

Ethics approval and consent to participate:

This study was granted ethical approval by Health Research Authority (HRA) London – City & East Research Ethics Committee (Reference: 19/LO/0356). All methods were conducted in accordance with the relevant guidelines and approved study documents. Informed consent was gained from all participants enrolled in this study.

Consent for publication:

Not applicable.

Availability of data and materials:

The data generated and analysed during the current study are not publicly available because ethical approval for their distribution has not been granted, nor have participants consented to it being made public.

Competing interests:

The authors declare that they have no competing interests.

Funding:

RBD was supported by the University of Southampton National Institute of Health Research Academic Clinical Fellowship Scheme. AAT is supported by the Medical Research Council (Clinical Research Training Fellowship MR/V002015/1).

Authors' contributions:

RBD, AAT, RCR, TV and CEJ conceived and designed the study. RBD implemented the study and conducted data analysis. RCR and CEJ provided supervision for the study. TV and CEJ provided supervision of the data interpretation. RBD prepared the manuscript. All authors reviewed, edited and approved the final manuscript.

Acknowledgements:

Not applicable.

For peer review only

Additional files

Additional material 1: Supplementary material 1

File name: lac-InterviewStudySupp1_20230323.docx

Description: Interview guide. Guide used during semi-structured interviews with participants.

Additional material 2: Supplementary material 2

File name: COREQchecklist_Lac_InterviewStudy20231101

Description: COREQ Checklist. Consolidated criteria for reporting qualitative studies (COREQ) checklist for the study "Qualitative interview study exploring the perspectives of pregnant women on participating in controlled human infection research in the UK".

For peer review only

References

1. McCarthy CR. Historical background of clinical trials involving women and minorities. Academic Medicine. 1994 Sep;69(9):695–8.

2. Kachikis A, Eckert LO, Englund JA. Chapter 1 - The history of maternal immunization. In: Leuridan EE, Nunes MC, Jones CE, editors. Maternal Immunization [Internet]. Academic Press; 2020 [cited 2022 Apr 26]. p. 3–24. Available from: https://www.sciencedirect.com/science/article/pii/B9780128145821000012

3. van der Zande ISE, van der Graaf R, Oudijk MA, van Delden JJM. Vulnerability of pregnant women in clinical research. J Med Ethics. 2017 Oct;43(10):657–63.

4. Council for International Organizations of Medical Sciences (CIOMS)x. International Ethical Guidelines for Health-related Research Involving Humans [Internet]. Fourth. Geneva: CIOMS publications; 2016 [cited 2022 May 16]. Available from: https://cioms.ch/publications/product/international-ethical-guidelines-for-health-related-research-involving-humans/

5. Widnes SF, Schjøtt J. Risk perception regarding drug use in pregnancy. American Journal of Obstetrics & Gynecology. 2017 Apr 1;216(4):375–8.

6. van der Zande ISE, van der Graaf R, Oudijk MA, van Delden JJM. A qualitative study on acceptable levels of risk for pregnant women in clinical research. BMC Med Ethics. 2017 Dec;18(1):35.

7. Adam MP, Polifka JE, Friedman JM. Evolving knowledge of the teratogenicity of medications in human pregnancy. American Journal of Medical Genetics. 2011;157(3):175–82.

8. Gomes MF, de la Fuente-Núñez V, Saxena A, Kuesel AC. Protected to death: systematic exclusion of pregnant women from Ebola virus disease trials. Reprod Health. 2017 Dec 14;14(Suppl 3):172.

9. Taylor MM, Kobeissi L, Kim C, Amin A, Thorson AE, Bellare NB, et al. Inclusion of pregnant women in COVID-19 treatment trials: a review and global call to action. The Lancet Global Health. 2021 Mar 1;9(3):e366–71.

10. Lyerly AD, Little MO, Faden R. The second wave: Toward responsible inclusion of pregnant women in research. Int J Fem Approaches Bioeth. 2008;1(2):5–22.

11. Strömmer S, Lawrence W, Rose T, Vogel C, Watson D, Botell JN, et al. Improving recruitment to clinical trials during pregnancy: a mixed methods investigation. Soc Sci Med. 2018 Mar;200:73–82.

12. Marbán-Castro E, Pons-Duran C, García-Otero L, Chen H, Herrera LB, Gil M del M, et al. Acceptability of Clinical Trials on COVID-19 during Pregnancy among Pregnant Women and Healthcare Providers: A Qualitative Study. IJERPH. 2021 Oct 13;18(20):10717.

13. Oude Rengerink K, Logtenberg S, Hooft L, Bossuyt PM, Mol BW. Pregnant womens' concerns when invited to a randomized trial: a qualitative case control study. BMC Pregnancy Childbirth. 2015 Sep 4;15:207.

For peer review only

14. Jamrozik E, Littler K, Bull S, Emerson C, Kang G, Kapulu M, et al. Key criteria for the ethical acceptability of COVID-19 human challenge studies: Report of a WHO Working Group. Vaccine. 2021 Jan 22;39(4):633–40.

15. Bambery B, Selgelid M, Weijer C, Savulescu J, Pollard AJ. Ethical Criteria for Human Challenge Studies in Infectious Diseases. Public Health Ethics. 2016 Apr 1;9(1):92–103.

16. Theodosiou AA, Laver JR, Dale AP, Cleary DW, Jones CE, Read RC. Controlled human infection with Neisseria lactamica in late pregnancy to measure horizontal transmission and microbiome changes in mother-neonate pairs: a single-arm interventional pilot study protocol. BMJ Open. 2022 May 18;12(5):e056081.

17. Theodosiou AA, Dorey RB, Read RC, Jones CE. The infant pharyngeal microbiomes: origin, impact and manipulation. Curr Opin Infect Dis. 2020 Dec;33(6):548–55.

18. Theodosiou AA, Dorey RB, Laver JR, Cleary DW, Read RC, Jones CE. Manipulating the infant respiratory microbiomes to improve clinical outcomes: A review of the literature. J Infect. 2021 Jun;82(6):247–52.

19. Dorey RB, Theodosiou AA, Read RC, Jones CE. The nonpathogenic commensal Neisseria: friends and foes in infectious disease. Curr Opin Infect Dis. 2019 Oct;32(5):490–6.

20. Cartwright KA, Stuart JM, Jones DM, Noah ND. The Stonehouse survey: nasopharyngeal carriage of meningococci and Neisseria lactamica. Epidemiol Infect. 1987 Dec;99(3):591–601.

21. Deasy AM, Guccione E, Dale AP, Andrews N, Evans CM, Bennett JS, et al. Nasal Inoculation of the Commensal Neisseria lactamica Inhibits Carriage of Neisseria meningitidis by Young Adults: A Controlled Human Infection Study. Clinical Infectious Diseases. 2015 May 15;60(10):1512–20.

22. Dale AP, Theodosiou AA, Laver JR, Roche EF, Hill AR, Gorringe A, et al. Controlled human infection with Neisseria lactamica induces B-cell responses that are cross-reactive with Neisseria meningitidis. The Journal of Immunology. 2020 May 1;204(1 Supplement):231.21-231.21.

23. Evans CM, Pratt CB, Matheson M, Vaughan TE, Findlow J, Borrow R, et al. Nasopharyngeal colonization by Neisseria lactamica and induction of protective immunity against Neisseria meningitidis. Clin Infect Dis. 2011 Jan 1;52(1):70–7.

24. Braun V, Clarke V. Using thematic analysis in psychology. Qualitative Research in Psychology. 2006 Jan 1;3(2):77–101.

25. Crozier SR, Robinson SM, Borland SE, Godfrey KM, Cooper C, Inskip HM, et al. Do women change their health behaviours in pregnancy? Findings from the Southampton Women's Survey. Paediatric and Perinatal Epidemiology. 2009;23(5):446–53.

26. Kim C, McEwen LN, Piette JD, Goewey J, Ferrara A, Walker EA. Risk Perception for Diabetes Among Women With Histories of Gestational Diabetes Mellitus. Diabetes Care. 2007 Sep 1;30(9):2281–6.

27. Hammer R, Inglin S. 'I don't think it's risky, but…': pregnant women's risk perceptions of maternal drinking and smoking. Health, Risk & Society. 2014 Jan 2;16(1):22–35.

For peer review only

28. Ortendahl MD PhD M, Näsman Fil lic P. Perception of Smoking-Related Health Consequences among Pregnant and Non-Pregnant Women. The American Journal on Addictions. 2007;16(6):521–7.

29. Robinson M, Pennell CE, McLean NJ, Tearne JE, Oddy WH, Newnham JP. Risk perception in pregnancy: Context, consequences, and clinical implications. European Psychologist. 2015;20(2):120–7.

30. Heaman M, Gupton A, Gregory D. Factors influencing pregnant women's perceptions of risk. MCN Am J Matern Child Nurs. 2004 Apr;29(2):111–6.

31. Reynolds WW, Nelson RM. Risk perception and decision processes underlying informed consent to research participation. Social Science & Medicine. 2007;65(10):2105–15.

32. Kost RG, Lee LM, Yessis J, Coller BS, Henderson DK. Assessing Research Participants' Perceptions of their Clinical Research Experiences. Clin Transl Sci. 2011 Dec;4(6):403–13.

33. Li M, Chapman GB. Why Do People Like Natural? Instrumental and Ideational Bases for the Naturalness Preference. Journal of Applied Social Psychology. 2012;42(12):2859–78.

34. Dibonaventura M daCosta, Chapman GB. Do decision biases predict bad decisions? Omission bias, naturalness bias, and influenza vaccination. Med Decis Making. 2008 Aug;28(4):532–9.

35. Meshaka R, Jeffares S, Sadrudin F, Huisman N, Saravanan P. Why do pregnant women participate in research? A patient participation investigation using Q-Methodology. Health Expect. 2017 Apr;20(2):188–97.

36. Montgomery GH, Erblich J, DiLorenzo T, Bovbjerg DH. Family and friends with disease: their impact on perceived risk. Prev Med. 2003 Sep;37(3):242–9.

37. Carolan MC. Towards understanding the concept of risk for pregnant women: some nursing and midwifery implications. J Clin Nurs. 2009 Mar;18(5):652–8.

38. Krubiner CB, Faden RR, Karron RA, Little MO, Lyerly AD, Abramson JS, et al. Pregnant women & vaccines against emerging epidemic threats: Ethics guidance for preparedness, research, and response. Vaccine. 2021 Jan 3;39(1):85–120.

39. Jasper JD, Goel R, Einarson A, Gallo M, Koren G. Effects of framing on teratogenic risk perception in pregnant women. Lancet. 2001 Oct 13;358(9289):1237–8.

40. Gross D, Fogg L. Clinical trials in the 21st century: the case for participant-centered research. Res Nurs Health. 2001 Dec;24(6):530–9.

41. Newburg SM, Holland AE, Pearce LA. Motivation of subjects to participate in a research trial. Applied Nursing Research. 1992 May 1;5(2):89–93.

For peer review only

Recommendations for research

- Redress the recruitment of pregnant women in research. Recent examples have shown that conducting research in pregnancy can be made safe and transparent. Furthermore, our study demonstrates that some pregnant women may be enthusiastic about engaging with research studies.
- Ensure that research is acceptable and feasible to pregnant women. Pregnant women should be engaged early in the design of research, through Patient and Public Involvement work, and detailed discussions held about study requirements, particularly the utility and scientific justification of each of these.
- Our data suggest that the method of intervention administration matters greatly to participants. Researchers should consider whether alternative methods of administration may better engage pregnant women in research.
- Recognise that the exclusion of pregnant women from research has a demonstrable impact on their medical care, and may in fact pose greater risk to them than study enrolment.

Recommendations for research. Four recommendations for how to improve equity of research for pregnant women.

158x92mm (300 x 300 DPI)

<u>*Neisseria lactamica* in pregnancy: an interview study - Interview guide</u>

Interview duration: 30 minutes (maximum 45 minutes)

This interview will take around 30 minutes of your time. Please stop me to ask questions if anything is not clear. <u>This is a guide and the interviewer has flexibility to explore unexpected areas raised by the study participants. The interview guide will be reviewed after the first few interviews and small changes may be made to content and order in light of the participants' responses.</u>

The aim of this interview is to understand what your thoughts are about a research study that we might potentially do in the future. There are no right or wrong answers. Your answers will help us to be better prepared for this study in the future.

With your permission, this interview will be audio recorded. All information you provide will be confidential, and any information that could identify you will be removed from all transcripts, and the audio will be deleted at the end of the study.

[*Note: allow prompting to clarify answers or to probe respondents thoughts further]*

Before we begin, do you have any questions or is anything not clear?

<u>[Section 1: Understanding of meningitis]</u>

<u>First of all, we would like to know what you understand about meningitis. Remember there are no right or wrong answers, we are just trying to gauge what people understand.</u>

1. Have you heard of meningitis before? Can you tell me what you understand about by the word "meningitis"?

    [Prompt: Do you worry about your child getting meningitis? Is this something you have thought about before? Do you know anyone how has had meningitis?]

Let me give you some information about meningitis to help with some further questions. Meningitis is an infection of the lining of the brain. It can be caused by bacteria that live in many people's noses and throats. Most of the time these bacteria do not cause any problems, but in some people they can, rarely, cause meningitis, and can make some people very unwell. To prevent meningitis, babies and teenagers are given vaccines. These vaccines protect against many of the bacteria that cause meningitis. However, we do not yet have vaccines that protect against all of the bacteria that cause meningitis. Furthermore, these vaccines are only given from 2 months of age, so they do not protect babies from birth.

We are hoping to carry out a research study in the future to see if we can better protect young babies from meningitis.

<u>[Section 2: Understanding of *Neisseria lactamica* and the microbiome]</u>

Neisseria lactamica is a type of 'good bacteria' that lives in the nose and throat. You might have heard about 'good or friendly bacteria' that can be found in some yoghurts. In healthy people with a normal immune system, 'good' bacteria do not cause any problems. Most people will carry Neisseria lactamica in their noses at some point in their lives, either as young children or as parents of young children.

For peer review only - http://bmjopen.bmj.com/site/about/guidelines.xhtml

We are thinking about doing a study where we would give Neisseria lactamica into the noses of pregnant women.

2. What is your first reaction to hearing this? How does this information make you feel?
3. How likely would you be to take part in a study which gave *Neisseria lactamica* into your nose whilst you were pregnant? How likely would you be to take part, with 1 being 'never' to 10 being 'definitely'? Can you explain why?

As I explained before, Neisseria lactamica is a type of 'good' bacteria that lives in the nose and throat. Having this bacteria in the nose and throat is common in childhood, with around 30% of children carrying it at any one time.

There is evidence that during outbreaks of meningitis, people who already have Neisseria lactamica in their noses are less likely to get meningitis. We have done studies in healthy adults showing that people who are given Neisseria lactamica into their noses were less likely to carry the bacteria that can cause meningitis. This is why we want to investigate Neisseria lactamica, to see whether we can protect people from meningitis, in particular young infants. We would aim to give pregnant women Neisseria lactamica into their noses at the end of pregnancy and see whether Neisseria lactamica is transferred to their babies noses in the few weeks after birth. We want to see if this changes the types of bacteria that babies carry in their noses, from less healthy types to more healthy types.

The human body is made up of more bacterial cells than human cells, the vast majority of which will never cause any problem. If someone has a severely weakened immune system, then any of these bacteria could theoretically cause infection, including Neisseria lactamica. However, there isn't any evidence that Neisseria lactamica causes infection in healthy adults.

<u>[Section 3: Acceptability the *Neisseria lactamica* study]</u>

4. After hearing more information, how likely is it that you would you take part in this study now? Can you rate how likely you would be to take part, with 1 being 'never' and 10 being definitely. Can you explain why?
   [Prompt: what concerns do you have? For example: Safety? If 10 people had already been given the bug would you accept it? What about if 1000 people or 10,000 people had?]

In the medical literature, there are three cases described which show Neisseria lactamica causing an infection in healthy children. These cases were reported between 1976 and 1978, and there have been no reported cases internationally since then.

5. Does this information change your mind about taking part in a research study involving *Neisseria lactamica*? Does this make the study seem more risky? What would reassure you?
6. What do you deem to be the most important risks?
   [Prompt: risk to you, risk to your baby?]
7. What other information would you require before considering participating? Which parts of the information you have heard, are most important to you, and why?
   [Prompt: Have we provided all the information that you would like to know?]
8. Having spoken about your concerns in the question before, if we provided all of this information, how likely are you now to participate in the study: 1 (never) -10 (definitely)?

For peer review only - http://bmjopen.bmj.com/site/about/guidelines.xhtml

[Prompt: Which information has helped you make up your mind?]

9. What information would most encourage you to participate in this study?
10. What information would put you off participating in this study?
11. We may not give *Neisseria lactamica* into the noses participants who are found already have it in their nose. If you were found to have low levels of *Neisseria lactamica* in your nose how would feel about:
    a. Being given the *Neisseria lactamica* spray into your nose to increase the amount that is present there?
    b. Not being given anything, but being followed-up as described above?

Study design of *Neisseria lactamica* study

12. How regularly would you deem it acceptable to come to clinic for this study during pregnancy (1-2 times, 3-4 times, 5-6 times, monthly)?
    [Prompt: would you be willing to be seen more regularly if we came to your home? How else could we facilitate participation in follow-up?]
13. How often would you a be prepared to attend follow-up with your baby?
    [Prompt: once at birth, two/three/ four/six times in the first year of life. How could we facilitate participation in follow-up?]
14. In addition to any sample collected as part of routine care, what samples would be acceptable to collect from:
    a. You - blood, throat swabs, nose wash, breast milk
    b. Infant - blood, nose swabs, throat swabs

Conclusion

15. Do you think this study is ethical?
    [Prompt: Do you think this study is acceptable to you? if yes, why? If no, what would need to change, and what other information would you need?]
16. Do you think this study should be done?
    Prompt: if yes, why? If no, why?
17. Is there anything else you would like to share or ask?

Finally, some questions about you:

18. How many weeks pregnant are you?
19. Have you had any children before? *(if no, move to question 20)*
20. How old are your children?
21. Have you participated in research before? During pregnancy?
22. Which ethnic group do you feel you belong to? [Prompt: use codes]

Thank you

For peer review only

For peer review only - http://bmjopen.bmj.com/site/about/guidelines.xhtml

Supplementary material 1. COREQ Checklist. Consolidated criteria for reporting qualitative studies (COREQ, (1)) checklist for the study “Qualitative interview study exploring the perspectives of pregnant women on participating in controlled human infection research in the UK”.

Domain 1: Research team and reflexivity

Personal Characteristics

1. Interviewer/facilitator – Which author/s conducted the interview or focus group?

RD

2. Credentials – What were the researcher's credentials? E.g. PhD, MD

BMBS, MSc, BMedSci(Hons), MRCPCH

3. Occupation –What was their occupation at the time of the study?

Paediatrician on an NIHR Academic Clinical Fellowship at University Hospital Southampton and University of Southampton

4. Gender – Was the researcher male or female?

Male

5. Experience and training – What experience or training did the researcher have?

RD has completed research degrees (MSc and BMedSci) and NIHR Good Clinical Practice. As well as experience in controlled human infection research.

Co-authors have experience in both clinical practice, controlled human infection research, and qualitative research.

Relationship with participants

6. Relationship established –Was a relationship established prior to study commencement?

No

7. Participant knowledge of the interviewer – What did the participants know about the researcher? e.g. personal goals, reasons for doing the research

Participants were made aware of the study aims and the researchers position and experience.

8. Interviewer characteristics – What characteristics were reported about the interviewer/facilitator? e.g. Bias, assumptions, reasons and interests in the research topic

Participants were informed that the results of the interview study would inform the design of the Lactamica 9 study (2) and that results would be analysed and published.

Domain 2: study design

For peer review only

Theoretical framework

9. Methodological orientation and Theory – What methodological orientation was stated to underpin the study? e.g. grounded theory, discourse analysis, ethnography, phenomenology, content analysis

Thematic analysis (3)

Participant selection

10. Sampling – How were participants selected? e.g. purposive, convenience, consecutive, snowball

Purposive sampling

11. Method of approach – How were participants approached? e.g. face-to-face, telephone, mail, email

Participants were approached in person an antenatal clinic in a maternity hospital and the interview took place face-to-face at that time.

12. Sample size – How many participants were in the study?

Twelve participants

13. Non-participation – How many people refused to participate or dropped out? Reasons?

There was not dropout during the study.

Setting

14. Setting of data collection – Where was the data collected? e.g. home, clinic, workplace

Clinic

15. Presence of non-participants – Was anyone else present besides the participants and researchers?

No

16. Description of sample – What are the important characteristics of the sample? e.g. demographic data, date

All participants were pregnant and so provided relevant insights into perceptions of risk of research in pregnancy.

Data collection

17. Interview guide – Were questions, prompts, guides provided by the authors? Was it pilot tested?

An interview guide was used (see supplementary material 1) which contained verbal information and question prompts for the interview. This was designed with input from a PPI group.

18. Repeat interviews – Were repeat interviews carried out? If yes, how many?

For peer review only

No

19. Audio/visual recording – Did the research use audio or visual recording to collect the data?

Audio recording of interviews was performed.

20. Field notes – Were field notes made during and/or after the interview or focus group?

No. Audio recordings of interviews were transcribed post hoc.

21. Duration – What was the duration of the interviews or focus group?

Interviews lasted 30 to 60 minutes.

22. Data saturation – Was data saturation discussed?

Participants were recruited until data saturation was reached as determined by RD, TV, and CJ.

23. Transcripts returned – Were transcripts returned to participants for comment and/or correction?

No

Domain 3: analysis and findings

Data analysis

24. Number of data coders – How many data coders coded the data?

Data was coded by RD and cross-checked by, and discussed with, between RD, TV, CJ.

25. Description of the coding tree – Did authors provide a description of the coding tree?

No

26. Derivation of themes – Were themes identified in advance or derived from the data?

Themes were derived from the data and are summaries in Table 2.

27. Software – What software, if applicable, was used to manage the data?

Data was manged using NVivo (version 1.6.1), see Methods.

28. Participant checking – Did participants provide feedback on the findings?

No

Reporting

29. Quotations presented - Were participant quotations presented to illustrate the themes / findings? Was each quotation identified? e.g. participant number

Yes, quotations are provided in the Results. Participant number and relevant demographic data provided.

For peer review only

30. Data and findings consistent – Was there consistency between the data presented and the findings?

Quotations are presented in sections of relevant themes within the Results. These are then explored in the context of existing literature within the Discussion.

31. Clarity of major themes – Were major themes clearly presented in the findings?

Themes are summaries in Table 2 at the beginning of the Results section.

32. Clarity of minor themes – Is there a description of diverse cases or discussion of minor themes?

Subthemes are also summarised in Table 2. These are then expanded upon in the relevant sections of the discussion with pertinent quotations provided.

References

1) Tong A, Sainsbury P, Craig J. Consolidated criteria for reporting qualitative research (COREQ): a 32-item checklist for interviews and focus groups. International Journal for Quality in Health Care. 2007;19(6):349–357.
2) Theodosiou AA, Laver JR, Dale AP, Cleary DW, Jones CE, Read RC. Controlled human infection with Neisseria lactamica in late pregnancy to measure horizontal transmission and microbiome changes in mother-neonate pairs: a single-arm interventional pilot study protocol. BMJ Open. 2022;12(5):e056081.
3) Braun V, Clarke V. Using thematic analysis in psychology. Qualitative Research in Psychology. 2006;3(2):77–101.

For peer review only
